# N-Terminal Acetylation by NatC Is Not a General Determinant for Substrate Subcellular Localization in *Saccharomyces cerevisiae*

**DOI:** 10.1371/journal.pone.0061012

**Published:** 2013-04-15

**Authors:** Henriette Aksnes, Camilla Osberg, Thomas Arnesen

**Affiliations:** 1 Department of Molecular Biology, University of Bergen, Bergen, Norway; 2 Department of Surgery, Haukeland University Hospital, Bergen, Norway; Centro de Investigación en Medicina Aplicada (CIMA), Spain

## Abstract

N-terminal acetylation has been suggested to play a role in the subcellular targeting of proteins, in particular those acetylated by the N-terminal acetyltransferase complex NatC. Based on previous positional proteomics data revealing N-terminal acetylation status and the predicted NAT substrate classes, we selected 13 suitable NatC substrates for subcellular localization studies in *Saccharomyces cerevisiae*. Fluorescence microscopy analysis of GFP-tagged candidates in the presence or absence of the NatC catalytic subunit Naa30 (Mak3) revealed unaltered localization patterns for all 13 candidates, thus arguing against a general role for the N-terminal acetyl group as a localization determinant. Furthermore, all organelle-localized substrates indicated undisrupted structures, thus suggesting that absence of NatC acetylation does not have a vast effect on organelle morphology in yeast.

## Introduction

N-terminal (Nt-) acetylation is a common protein modification that involves the transfer of an acetyl group from acetyl-CoA to the α-amino group of the very first amino acid of a polypeptide. The reaction is catalyzed by N-terminal acetyltransferases (NATs), comprising a group of five enzymes in yeast, NatA-E, that differ in their substrate specificity [1]. Despite the fact that most proteins undergo Nt-acetylation [2], the molecular and cellular implications of this modification are only just beginning to unveil.

Several functional effects of Nt-acetylation have been demonstrated, typically by studying individual substrate proteins in the absence of their cognate NAT. Examples of substrates and processes that are affected by absence of Nt-acetylation, reviewed in [3] include actin and tropomyosin affecting normal regulation of actin filaments [4]–[6]; Tfs1 affecting its inhibition of CPY [7]; Sir3 affecting its gene silencing [8]–[10]; and Ubc12 affecting its binding to and activation of Dcn1 [11]. However, based on current evidence, it is difficult to infer a *general* function of such Nt-acetyl groups. Of more wide-ranging functions of Nt-acetylation affecting several substrate proteins, three have been presented thus far: the Nt-acetyl group has been shown to function (i) in the formation of an N-terminal degradation signal [12]; (ii) in preventing post-translational translocation through the ER membrane [13]; and (iii) in protein-protein interactions and complex formation of which some of the abovementioned NAT substrates are examples. It is possible that also subcellular targeting belongs to this category of more general implications of Nt-acetylation, as this has been shown for several proteins.

The role of Nt-acetylation in subcellular targeting has been raised by findings suggesting that the Nt-acetyl group represents an important factor for protein targeting to membranes. This suggestion is largely based on studies in yeast *Saccharomyces cerevisiae* showing that certain NatC substrates lose their defined subcellular localization upon deletion of the gene encoding the NatC catalytic subunit, Naa30 (Mak3). The Golgi proteins Arl3 [14], [15] and Grh1 [16] mislocalize to the cytoplasm; and the inner nuclear membrane protein Trm1-II shifts to a nucleoplasmic localization [17] in cells lacking *NAA30*. NatC acetylates methionine-starting hydrophobic N-termini like Met-Leu-, Met-Phe-, Met-Ile- and Met-Tyr- [18]–[20]. Thus, the acetyl group could possibly act as a membrane interaction-stabilizator for the relatively hydrophobic N-termini that are subjected to NatC-mediated acetylation.

Here we further investigate the relationship between Nt-acetylation and protein subcellular localization. We address whether there could be a general role for NatC as a localization determinant by studying the GFP localization pattern of 13 likely NatC substrates in yeast cells lacking a genomic copy of *NAA30*.

## Results

### Selection of Candidates

Based on i) predicted substrate classes, where Met-Leu-, Met-Phe-, Met-Ile- and Met-Tyr- are potential NatC substrates [1], ii) previous positional proteomics data confirming that these particular proteins are Nt-acetylated in *S. cerevisiae* [2], [21], [22] and Arnesen et al., unpublished, iii) subcellular localization patterns, and iv) availability of GFP-tagged yeast strains, we selected 13 NatC substrates of interest (Table 1). Our study additionally included two of the previously published NatC substrates, Arl3 and Trm1-II, whose localization has been shown to be dependent on Naa30.

**Table 1:** NatC substrates included in this study.

Protein	Subcellular localization^1^	Protein type/Subcellular targeting mechanism	Nt-sequence	N-terminal acetylation status
Arl3	Golgi	Amphipathic α-helix; protein-protein interaction [14]	MFHLVK	Fully acetylated [14], [15]
Trm1-II	Inner nuclear membrane	Peripherally associated; INM targeting/tetheringmotif [23]	MLKAAI	Fully acetylated [17]
Sec18	Golgi/early Golgi	Recruited by Sec17 [24]; [25]	MFKIPG	Fully acetylated [22]
Sly41	ER/ER to Golgi	Predicted multipass transmembrane [26]; [27]	MIQTQS	Fully acetylated [21]
Nup157	Nuclear membrane	Component of the nuclear pore complex [28]	MYSTPL	Fully acetylated [2]
Rrn11	Nucleolus	Subunit of core factor (CF) complex [29],recruited in an SPT15/TBP-dependentmanner [30]	MFEVPI	Partially acetylated, Arnesen *et al*., unpublished
Rfc2	Nucleus	Subunit of the DNA-binding Replicationfactor C (RF-C) [31]	MFEGFG	Fully acetylated [22]
Ymr31	Mitocondrial lumen	N-terminal mitochondrial matrix targetingsequence [32]	MIATPIR	Partially acetylated [22]
Pda1	Mitochondria	N-terminal mitochondrial matrix targetingsequence [32]	MLAASF	Fully acetylated [22]
Glr1	Cytosol/Mitochondria/Nucleus	Mitochondrial targeting sequence present in geneproduct from 1^st^ TIS. 2^nd^ TIS lacksthis sequence and is cytosolic. [33]	MLSATK	Fully acetylated [21]
Tma20	Cytosol	Associates with ribosomes; putativeRNA-binding domain [34]	MFKKFT	Fully acetylated [22]
Lrg1	Bud neck/Cell periphery/Cytosol	N-terminal LIM domains required for localizationto sites of growth [35]	MIQNSA	Fully acetylated [22]
Bem1	Bud neck/Cell periphery	Recruited to bud site by Bud1 [36]	MLKNFK	Fully acetylated [21]
Pxl1	Sites of polarized growth	C-terminal LIM domain required for localizationto sites of polarized growth [37]	MYNSIY	Fully acetylated [22]
Tgl1	Lipid particle membranes	Transmembrane; proposed TypeI with cytosolicC-terminal and lumenal N-termial [38]	MYFPFL	Partially acetylated, Arnesen *et al*., unpublished

1 As described by Huh *et al*. [23] and observed in this study.

### Strain Generation and Verification

GFP collection strains [39] were obtained for all candidates and *NAA30* was deleted using homologous recombination. *NAA30*-deletion was verified in all strains by means of genomic PCR, demonstrated by the presence of the *kanMX4* selection cassette at the *NAA30* locus.

**Figure 1:**
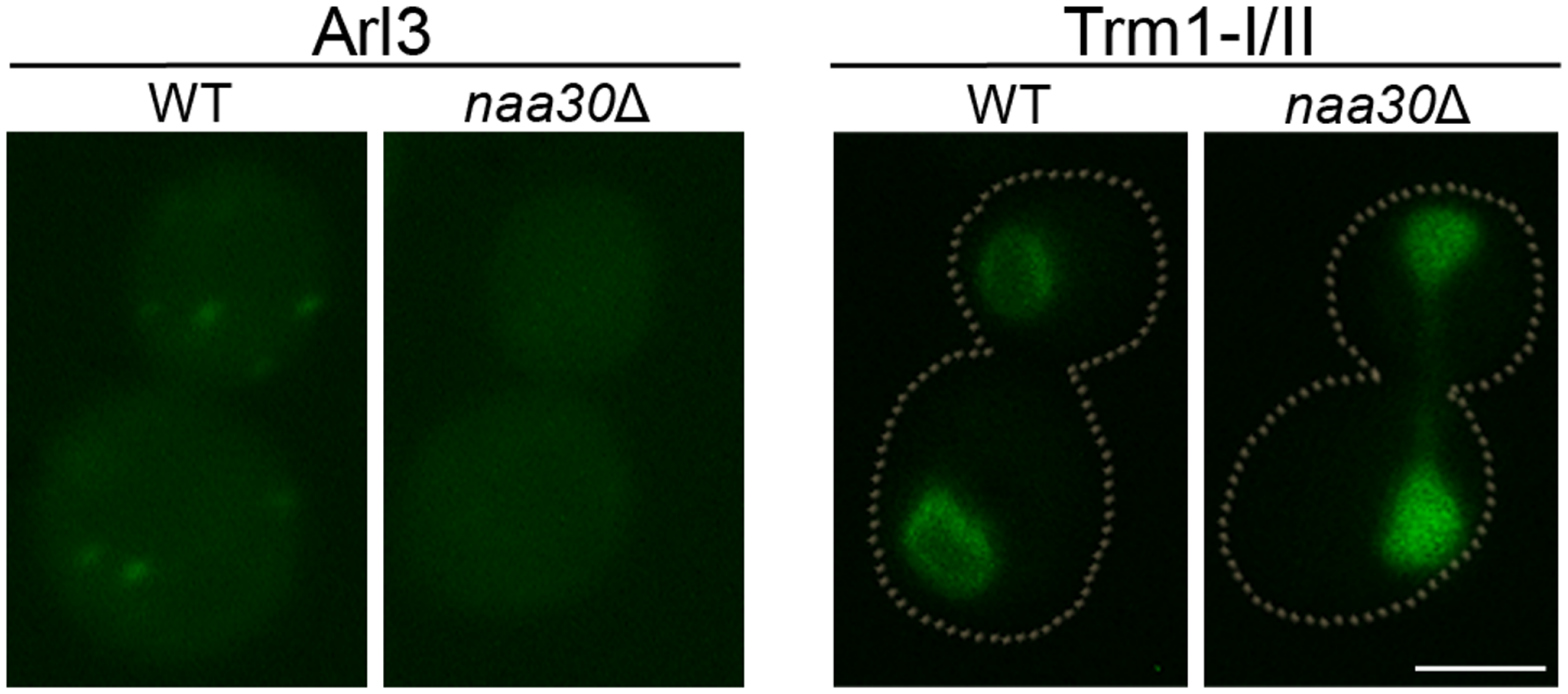
Subcellular localization of Arl3 and Trm1 in wild type and *naa30*Δ cells. The defined subcellular localization of Arl3 and Trm1-II was lost in *naa30*Δ cells. Arl3 and Trm1-GFP-fusion proteins are endogenously expressed. Trm1-GFP, gives rise to both forms (I and II) of Trm1, resulting from alternative translation initiation sites. See text for details. Scale bar 2 µm.

Deletion of *NAA30*, which encodes the NatC catalytic subunit, gave the expected loss of localization from the Golgi and inner nuclear membrane for Arl3 and Trm1-II, respectively (Figure 1, Figure S1 and Figure S2). Arl3 shifted from a clear punctuate pattern to a diffuse cytosolic pattern, as previously reported [14], [15]. In the case of Trm1, two translation initiation sites exist (I and II). Thus in our genomically tagged strain two C-terminally GFP-tagged proteins are produced: Trm1-I localizing to the nucleoplasm and Trm1-II to the inner nuclear membrane. Nevertheless, we were able to observe the same Naa30-dependent localization of Trm1-II as Murthi and Hopper [17]. The wild type strain had both a nucleoplasmic and a nuclear membrane pattern, whereas the nuclear membrane signal was absent and the nucleoplasmic signal increased in the strain lacking *NAA30* (Figure 1 and Figure S2).

### Maintained Subcellular Localizations of NatC-substrate Candidates in *naa30*Δ Cells

**Figure 2:**
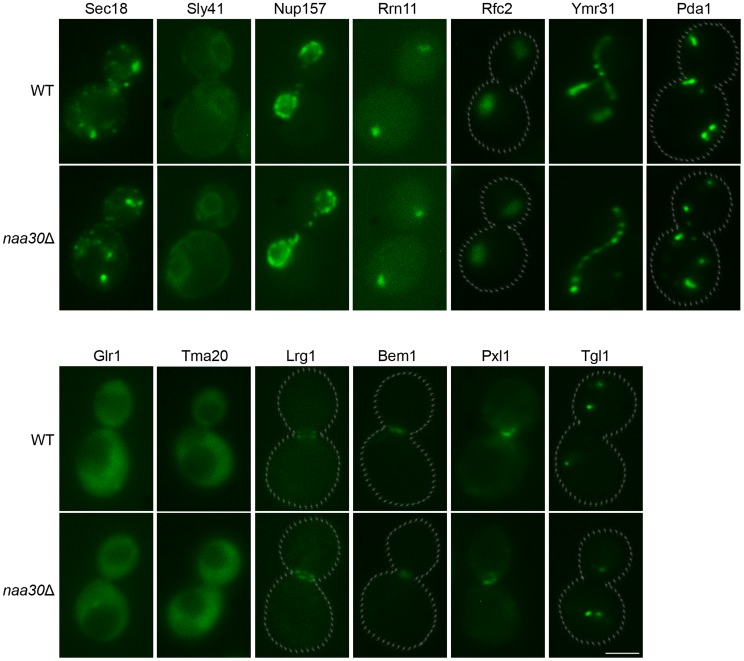
Subcellular localizations of putative NatC substrates in wild type and *naa30*Δ cells. GFP-localization patterns for all 13 candidates were unaffected by *NAA30*-deletion. The Golgi localized Sec18; ER localized Sly41; nuclear membrane localized Nup157; nucleolus localized Rrn11; nucleus localized Rfc2; mitochondrial Ymr31 and Pda1; cytosolic and nuclear Glr1; cytosolic Tma20; bud neck localized Lrg1, Bem1 and Pxl1; and a protein localized to lipid particle membranes, Tgl1, were all unaffected in their subcellular localization pattern as investigated by fluorescence microscopy. GFP-fusion proteins are endogenously expressed. Scale bar 2 µm.

**Figure 3:**
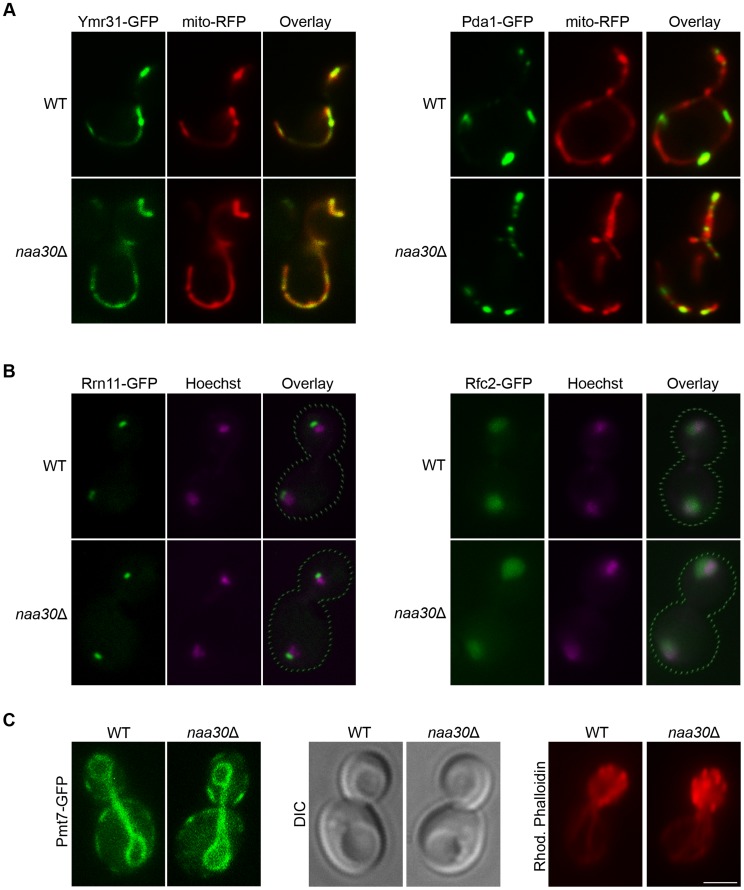
Organelle markers verified maintained substrate localization and suggested unaffected organelle morphology in the *naa30*Δ strain. The mitochondrial marker mito-RFP (A) confirmed the maintained mitochondrial localizations of Ymr31 and Pda1; and the nuclear DNA binding dye Hoechst (B) confirmed the maintained nuclear localizations of Rrn11 and Rfc2 in the *naa30*Δ cells. (C) The ER protein Pmt7 demonstrated the assumingly intact structure of this organelle; the DIC image demonstrated the intact vacuole structure; and Rhodamine Phalloidin staining of fixed cells suggested intact actin cytoskeleton in the cells lacking *NAA30*. Scale bar 2 µm.

As opposed to Arl3 and Trm1-II, none of the 13 herein investigated NatC substrate candidates mislocalized in the *NAA30*-deleted cells (Figure 2 and Figures S3–15). Localizations were maintained for the Golgi and ER proteins Sec18 and Sly41 (Figure 2, Figure S3 and Figure S4); the nuclear membrane protein Nup157 (Figure 2 and Figure S5); the nucleolus protein Rrn11 (Figure 2 and Figure S6); the nuclear protein Rfc2 (Figure 2 and Figure S7); the mitochondrial proteins Ymr31 and Pda1 (Figure 2, Figure S8 and Figure S9); the cytosolic and nuclear Glr1 (Figure 2 and Figure S10); the cytosolic Tma20 (Figure 2 and Figure S11); the bud neck proteins Lrg1, Bem1 and Pxl1 (Figure 2, Figures S12–S14); and a protein localized to lipid particle membranes, Tgl1 (Figure 2 and Figure S15). For the mitochondrial and nuclear proteins, the maintained subcellular localization was strengthened by supportive experiments using the mitochondrial marker mito-RFP (Figure 3A); the vital stain MitoTracker Red (data not shown); and the nuclear DNA stain Hoechst (Figure 3B). Some of the proteins had multiple subcellular localizations, thus complicating the microscopic analysis. For Glr1, which localizes to cytosol, mitochondria and nucleus, we were able to observe maintained localization to the cytosol and nucleus but the mitochondrial localization could not be visualized in either wild type or *NAA30*-deleted cells. Among the bud-neck proteins the localizations varied depending on cell cycle status. In addition to the bud-neck localizations showed for Lrg1, Bem1 and Pxl1, these proteins also localized to sites of polarized growth near *de novo* and progressed budding sites in wild type as well as *naa30*Δ cells (Figures S12–S14). Pxl1 also had cytoplasmic localization and appeared accumulated at bud-sites only at particular shorter periods of the budding cycle. Again, this characteristic was observed in wild type and *naa30*Δ cells alike (Figure S14). In summary, a broad selection of proteins, representing all currently known subtypes of NatC-type substrates as well as various subcellular localizations, were unaffected in their localization pattern in the absence of the NatC catalytic subunit Naa30.

Taking an alternative perspective on our data, the GFP-tagged proteins can also be viewed as organellar markers for the various subcellular localizations represented among the 13 candidates. Thus, our data also suggests the intactness of these organelles and subcellular structures as judged by the unchanged shape, number, size and distribution in the cell. No disruption of the nuclear membrane; endoplasmic reticulum; Golgi apparatus; mitochondria; or bud neck was observed. In the case of the endoplasmic reticulum (which was not fully visualized by Sly41-GFP due to low abundance) the morphological intactness of the organelle, including the cortical part of the ER was demonstrated by another ER protein, Pmt7-GFP (Figure 3C). Vacuoles also appeared unaffected by *NAA30*-deletion, as judged by the DIC image (Figure 3C). Intact actin cytoskeletal structure was demonstrated in fixed cells using Rhodamine Phalloidin (Figure 3C).

Since many membrane proteins are important for organellar regulation and maintenance, their mislocalization often cause abnormal organelle morphology [40]. Therefore, the unaffected morphology of these organellar structures in the absence of Naa30 further supports the notion that Nt-acetylation does not have an immense effect on protein localization.

## Discussion

Our data suggests that N-terminal acetylation by NatC does not generally determine the subcellular localization of its substrates. Of 15 putative NatC substrates analyzed in the current study, only the two previously published proteins, Arl3 and Trm1-II mislocalized in cells lacking *NAA30*. Although the few previously described examples of acetylation-facilitated membrane targeting provides important evidence of the functional importance of Nt-acetylation, it is possible that they merely are examples of single proteins or a minor subgroup of proteins that need their N-terminal acetyl group for correct localization.

In addition to the N-terminal acetyl group, the localization of the two Golgi proteins Arl3 and Grh1 also depends on their N-terminal amphipathic helix; and among predicted NatC substrates, these two are the only known exemplars that have this combination of features [16], thus indicating that these may represent individual cases where the N-terminal acetyl group determines the membrane localization.

It should also be noted that the three known examples of Nt-acetylation-dependent membrane localization are all suggested to occur through interactions with membrane proteins - Arl3 with Sys1 [14], [15]; Grh1 with Bug1 [16]; and Trm1-II with Ice2 or an inner nuclear membrane tether [17]. Thus perhaps a more suitable collective term for these effects of Nt-acetylation is protein-protein interactions, rather than protein-membrane interactions. In fact, several of the effects mentioned in the introduction are caused by disrupted protein-protein interactions in NAT-deficient cells.

A similar study of substrate localization effects have been performed for NatB using *NAT3* (*NAA20*, NatB catalytic subunit) deleted cells [41]. Here, eight nuclear proteins (Ioc3, Swi3, Spt8, Gis1, Yhp1, Pgd1, Dst1, and Syf2); two spindle-pole body proteins (Kar9 and Kar3); a nuclear periphery protein (Thp1); and two bud neck proteins (Mlc2 and Bud3) did not show any difference in localization between wild type and *naa20*Δ cells. Together, the NatB study and the present NatC work provide evidence against a *general* function of the Nt-acetyl group in protein localization. The observations that proteins destined for post-translational ER-translocation are not subjected to Nt-acetylation and that Nt-acetylation may prevent proteins from this translocation [13] represent a distinct concept that still holds independent of the herein presented data.

It should also be mentioned that for some of the candidates we observed either slightly increased or decreased intensity of the GFP-signal in the *naa30*Δ cells (data not shown). However, this was not further verified or followed up in this study. It is possible that these changes in GFP-intensity reflect a role of Nt-acetylation in protein translation or stability, as described previously [12], [42]. It is also possible that partial targeting defects could be the cause of these weak changes in protein abundance. As such, we cannot completely rule out the possibility that there were some minor effects on protein targeting that we were unable to observe in this study.

Our data may also indicate that several yeast organelles are not grossly altered in their morphology in the absence of NatC-acetylation. Previous studies have revealed some morphological effects on the *S. cerevisiae* plasma membrane structures eisosomes and MCC compartments after *NAA30* deletion [43], [44]. Also, siRNA-mediated h*NAA30*-depletion in mammalian cells causes scattering of the cis-Golgi (Starheim K. *et al*., submitted). Thus it could be that the implied mammalian role of Naa30 in organizing the Golgi apparatus is not pertinent in *S. cerevisiae*. However, the Golgi apparatus in *S. cerevisiae*, is less organized and does not typically assemble into stacks; instead, early and late cisternae are dispersed throughout the cytoplasm [45], [46]. As a consequence, irregular Golgi structure might be more difficult to discern in *S. cerevisiae* and thus we cannot exclude Golgi-associated effects beyond the detection limit of this study. Possible effects on Golgi or the secretory pathway could be further explored by future proteomics analyses of subcellular and extracellular fractions of *naa30*Δ yeast and/or in depth morphological studies using electron microscopy.

## Materials and Methods

### Yeast Strains

A complete list of all strains used in this study can be found in Table S1. All GFP strains are derivatives of BY4741 (*Mata; his3Δ1; leu2Δ0; met15Δ0; ura3Δ0*) from the yeast GFP collection (Invitrogen). Yeast gene deletion was done by standard PCR-based methods. *NAA30*-*kanMX4* deletion cassette was obtained from strain BY4742 YPR051w*Δ*::*kanMX4* (Acc: Y15470) of the yeast deletion collection (EUROSCARF). Cassette amplification primers were y*NAA30* −74F (5′-CATGAAGAACAAAGTTTCAC-3′) and 639R (5′-CCTCTTTTCTCTACTGCC-3′). Disruption of *NAA30* through homologous recombination was verified by genomic PCR using primers y*NAA30* −144F (5′-CTACCAAGAAACCGGGTAGC-3′) and 672R (5′-GAAACTTGCTTTATTATCTCTC-3′). Wild type and deletion strains of BY4741 YMR31-GFP and PDA1-GFP were transformed with the plasmid pYX142-*mtRFPm* which was a kind gift from Janet M. Shaw and Koji Okamoto [47].

### Yeast Cultivation and Preparation for Imaging

All yeast cultivation was done in YPD or SC-Leu medium. In preparation for imaging of live GFP-strains, yeast were diluted back from over night culture and allowed to grow for 4 hours until early exponential phase (OD_600_ 0.8–1.2). At this point, cells were washed three times and dissolved in PBS. A 2-µl drop of cell suspension was placed between an objective glass and a coverslip.

The yeast vital stains MitoTracker Red (CMXRos) and Hoechst (33342) (both from Invitrogen) were added to the growing cells at a concentration of 0.2 µg/ml and 1 µg/ml, respectively, 15–20 min prior to washing and imaging. Phalloidin staining of actin cytoskeleton was performed on fixed cells according to the Cold Spring Harbor protocol [48].

### Fluorescence Microscopy

Imaging was performed on a Leica DMI6000 B widefield microscope equipped with a Leica DC500 camera and a 100×1.4 NA oil objective in addition to a 2× magnification lens. GFP-tagged proteins were visualized in live yeast cells using excitation bandpass filter 430–510 nm and bandpass suppression filter 475–550 nm. RFP, Rhodamine and MitoTracker Red were imaged with bandpass filter 520–600 nm and suppression filter 570–720. Hoechst was imaged with excitation bandpass filter 340–380 nm and longpass suppression filter 425 nm.

Settings for intensity and exposure time of the excitation light used for GFP were optimized for each set of wild type and deletion GFP-strains, but were typically maximum intensity for 1000–1500 ms. All images were acquired using Leica LAS AF software and post-acquisition processing was performed in Adobe Photoshop CS5. The DIC channel was used to obtain the cell border indicated with a dashed line in Figures 1–3.

Each pair of WT and *naa30*Δ strain was imaged on two separate experimental days and at least 30 images, containing 5–20 cells per image, were the basis for the determination of the localization pattern. The representative phenotypes are shown.

## Supporting Information

Figure S1 **Full-field view of Arl3-GFP cells.** The Golgi localization of Arl3 in wild type cells (A) was lost in *naa30*Δ cells (B).
(PDF)

Figure S2 **Full-field view of Trm1-GFP cells.** The inner nuclear membrane localization of Trm1-II in wild type cells (A) was lost in *naa30*Δ cells (B) where Trm1-II accumulated in the nucleoplasm.
(PDF)

Figure S3 **Full-field view of Sec18-GFP cells.** The early Golgi localization of Sec18 in wild type cells (A) was maintained in *naa30*Δ cells (B).
(PDF)

Figure S4 **Full-field view of Sly41-GFP cells.** The ER localization of Sly41 in wild type cells (A) was maintained in *naa30*Δ cells (B).
(PDF)

Figure S5 **Full-field view of Nup157-GFP cells.** The nuclear pore localization of Nup157 in wild type cells (A) was maintained in *naa30*Δ cells (B).
(PDF)

Figure S6 **Full-field view of Rrn11-GFP cells.** The nucleolus localization of Rrn11 in wild type cells (A) was maintained in *naa30*Δ cells (B).
(PDF)

Figure S7 **Full-field view of Rfc2-GFP cells.** The nuclear localization of Rfc2 in wild type cells (A) was maintained in *naa30*Δ cells (B).
(PDF)

Figure S8 **Full-field view of Ymr31-GFP cells.** The mitochondrial localization of Ymr31 in wild type cells (A) was maintained in *naa30*Δ cells (B).
(PDF)

Figure S9 **Full-field view of Pda1-GFP cells.** The mitochondrial localization of Pda1 in wild type cells (A) was maintained in *naa30*Δ cells (B).
(PDF)

Figure S10 **Full-field view of Glr1-GFP cells.** The cytosolic and nuclear localization of Glr1 in wild type cells (A) was maintained in *naa30*Δ cells (B). The described mitochondrial localization could not be distinguished in either wild type or *naa30*Δ cells.
(PDF)

Figure S11 **Full-field view of Tma20-GFP cells.** The cytosolic localization of Tma20 in wild type cells (A) was maintained in *naa30*Δ cells (B).
(PDF)

Figure S12 **Full-field view of Lrg1-GFP cells.** The bud-neck localization of Lrg1 in wild type cells (A) was maintained in *naa30*Δ cells (B).
(PDF)

Figure S13 **Full-field view of Bem1-GFP cells.** The bud-neck localization of Bem1 in wild type cells (A) was maintained in *naa30*Δ cells (B).
(PDF)

Figure S14 **Full-field view of Pxl1-GFP cells.** The localization of Pxl1 to sites of polarized growth in wild type cells (A) was maintained in *naa30*Δ cells (B).
(PDF)

Figure S15 **Full-field view of Tgl1-GFP cells.** The lipid particle localization of Tgl1 in wild type cells (A) was maintained in *naa30*Δ cells (B).
(PDF)

Table S1 **Yeast strains used in this study.**
(PDF)
