# Supplementary figures and images for: N-Terminal Acetylation by NatC Is Not a General Determinant for Substrate Subcellular Localization in Saccharomyces cerevisiae
Source: PLoS One. 2013 Apr 15;8(4):e61012. doi: 10.1371/journal.pone.0061012 (PMC3626706; doi:10.1371/journal.pone.0061012)

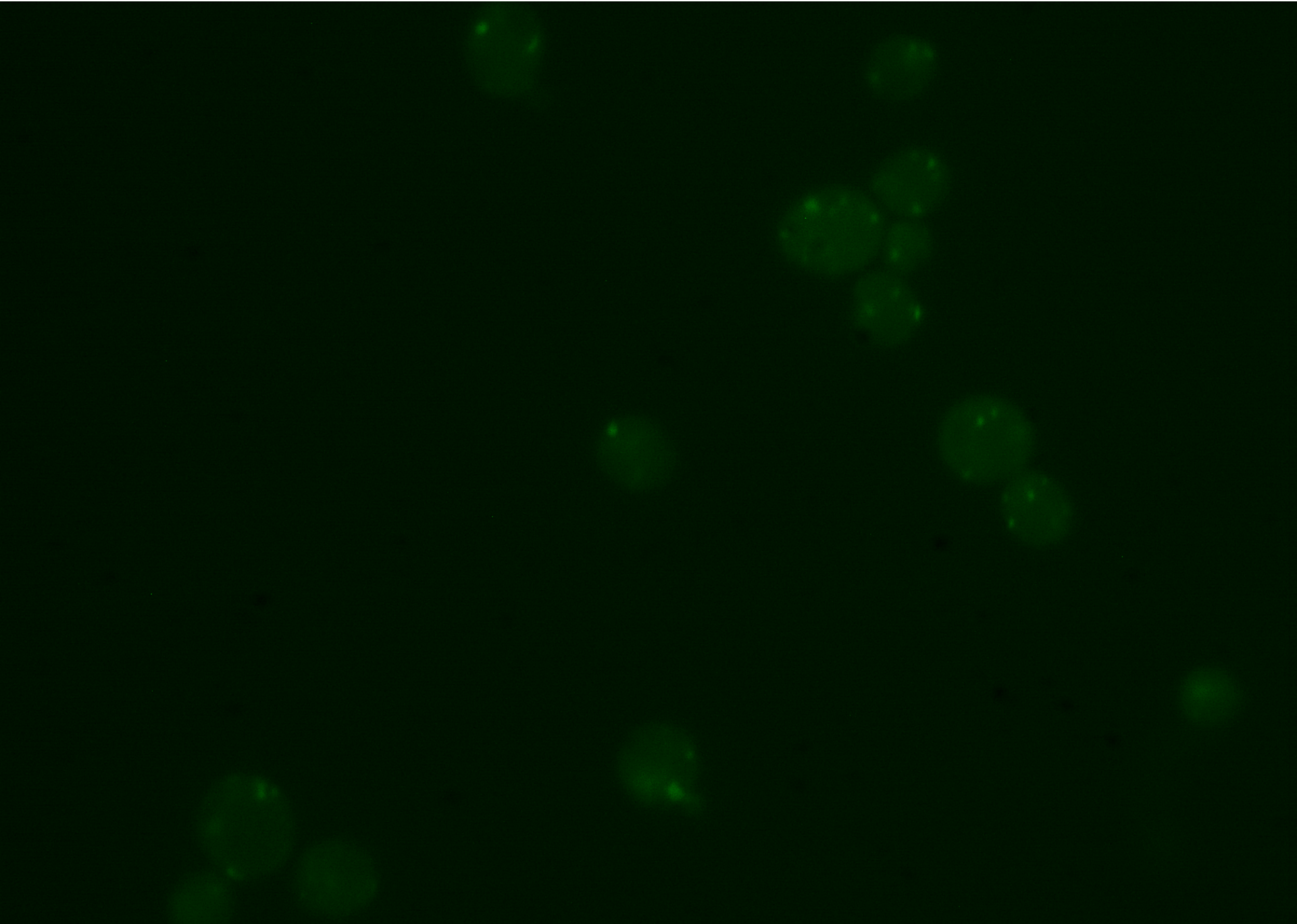

S1A. Arl3-GFP in yNAA30 WT

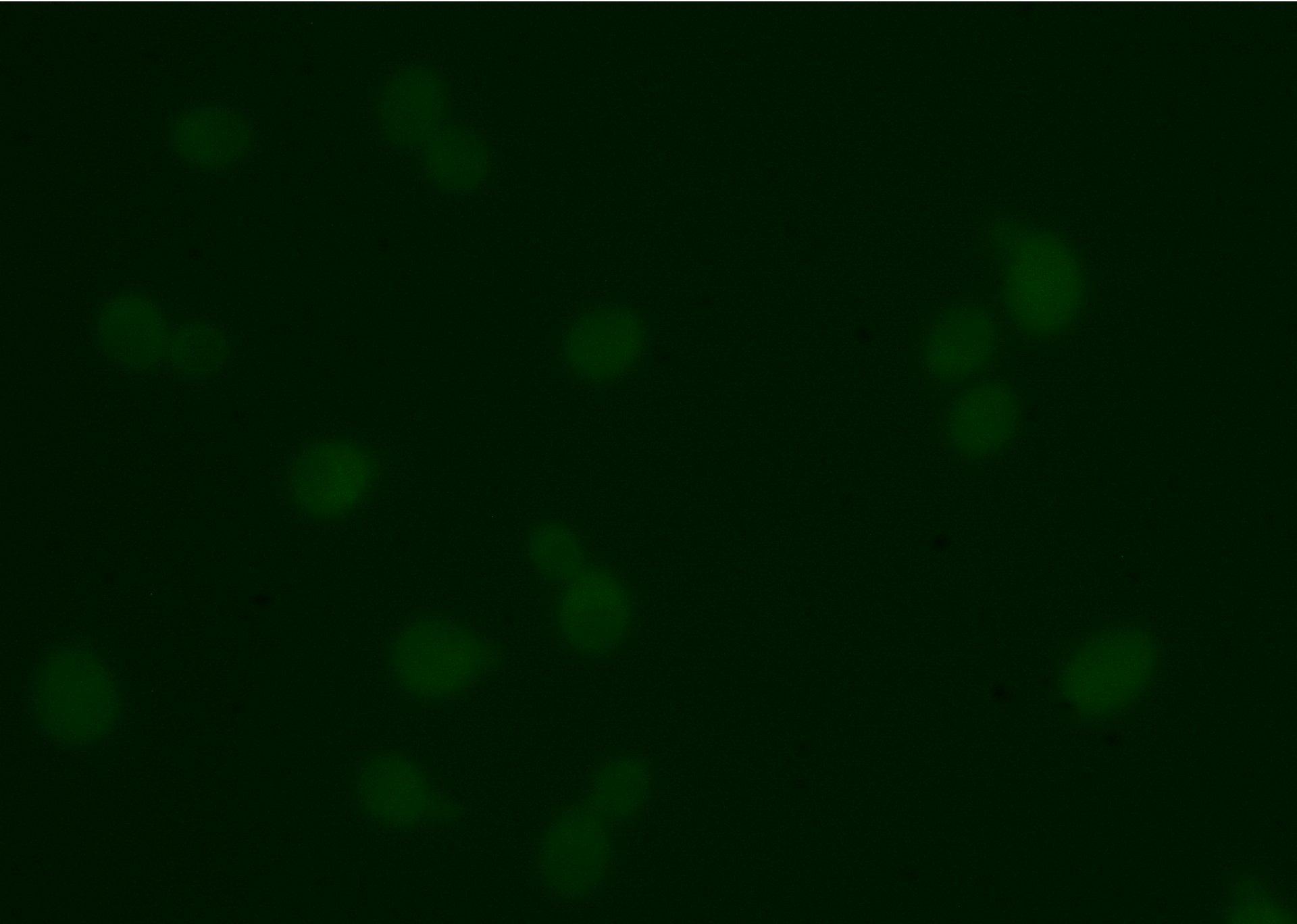

S1B. Arl3-GFP in *yna30Δ*

Supplement: Figure S1 — Full-field view of Arl3-GFP cells. The Golgi localization of Arl3 in wild type cells (A) was lost in naa30Δ cells (B). (PDF) [file pone.0061012.s001.pdf]

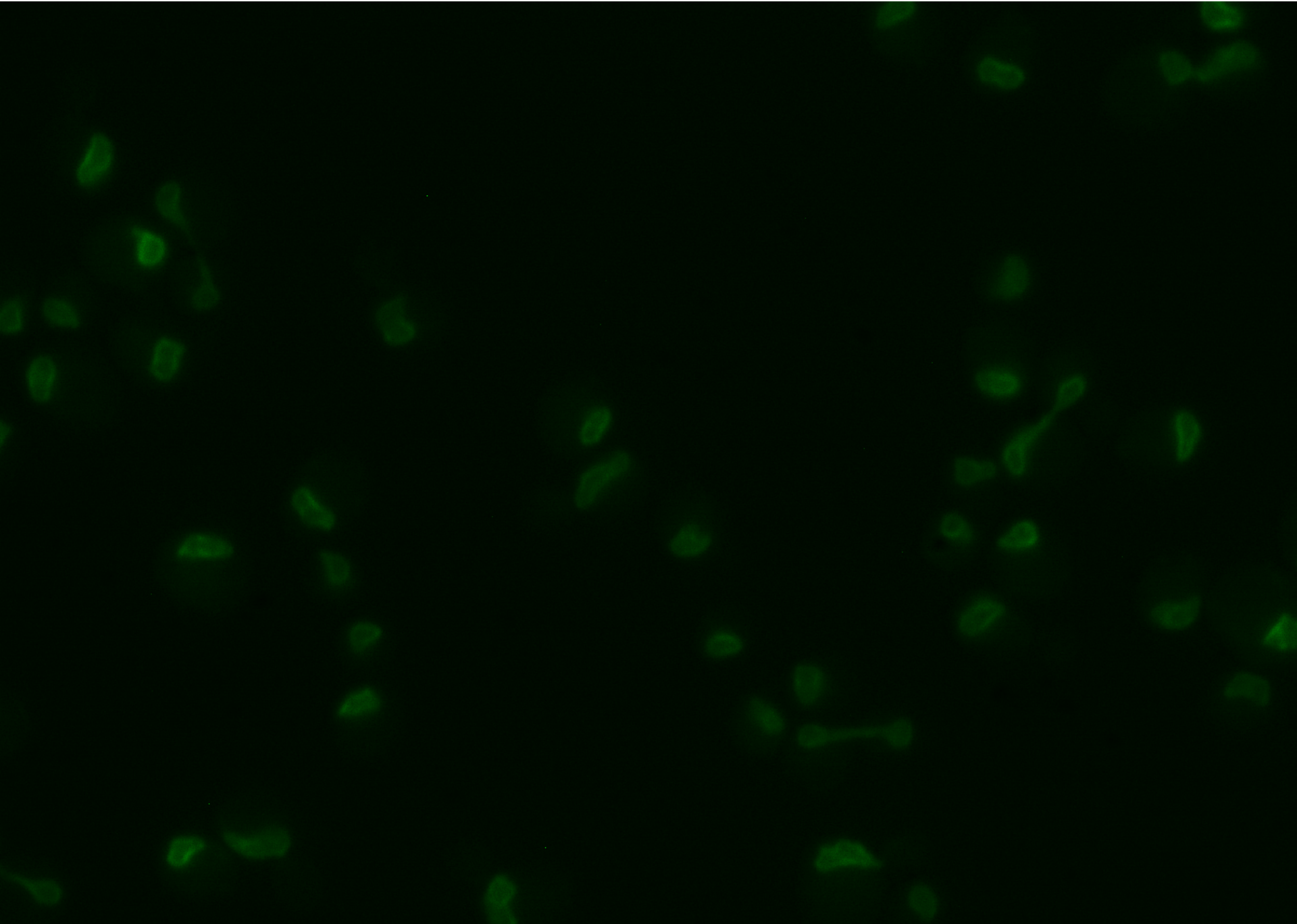

S2A. Trm1-GFP in yNAA30 WT

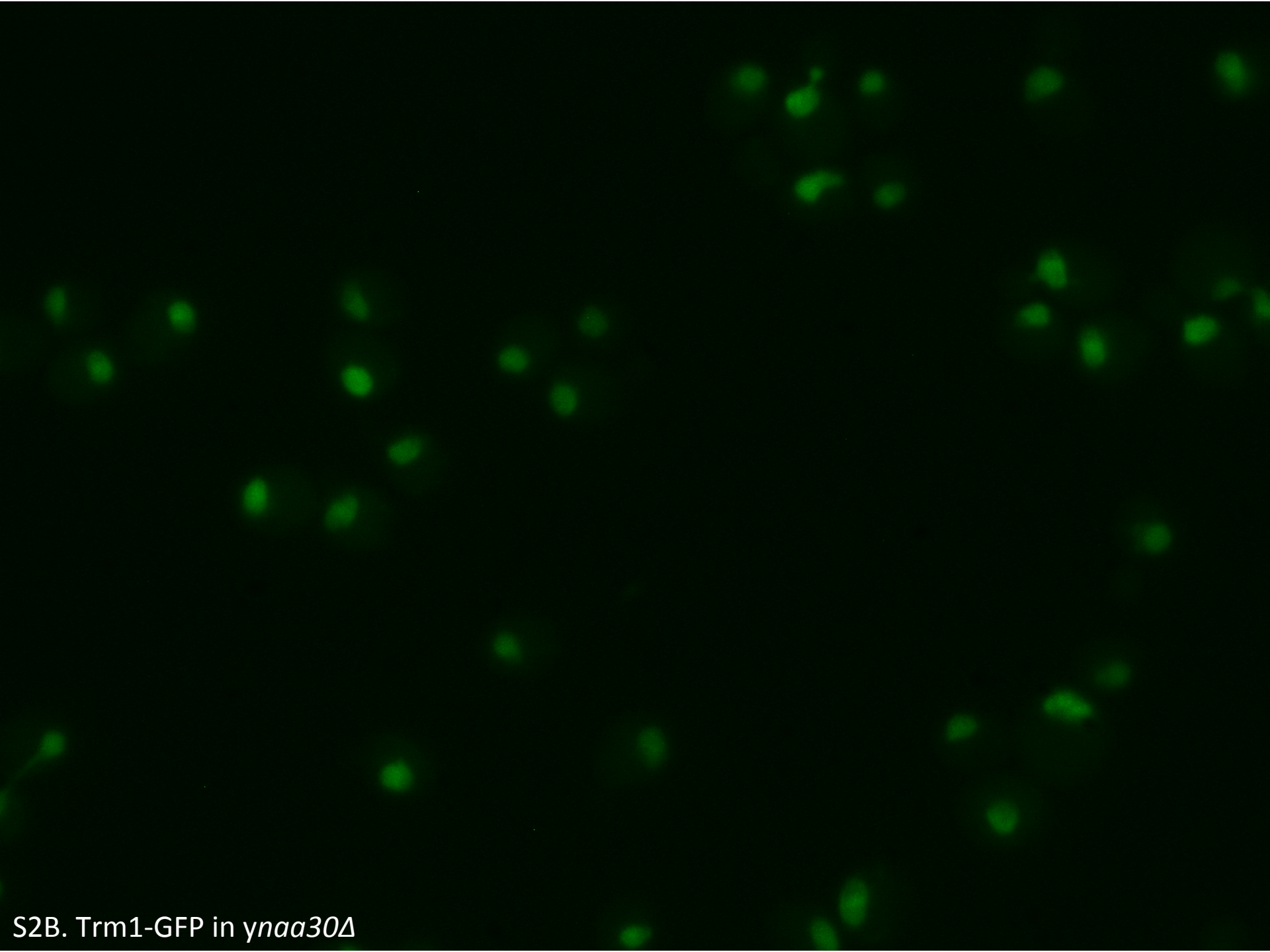

S2B. Trm1-GFP in *yna30Δ*

Supplement: Figure S2 — Full-field view of Trm1-GFP cells. The inner nuclear membrane localization of Trm1-II in wild type cells (A) was lost in naa30Δ cells (B) where Trm1-II accumulated in the nucleoplasm. (PDF) [file pone.0061012.s002.pdf]

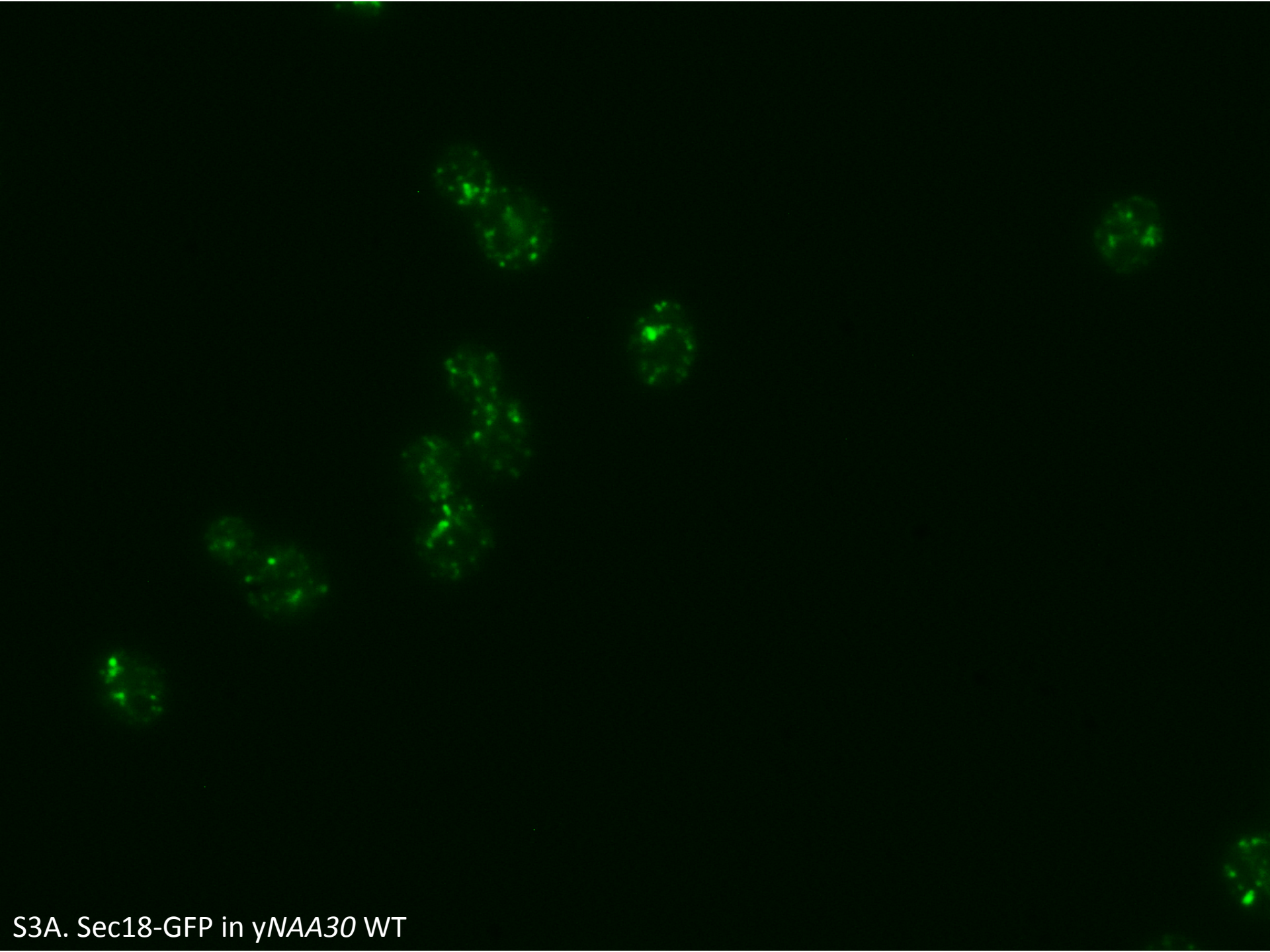

S3A. Sec18-GFP in yNAA30 WT

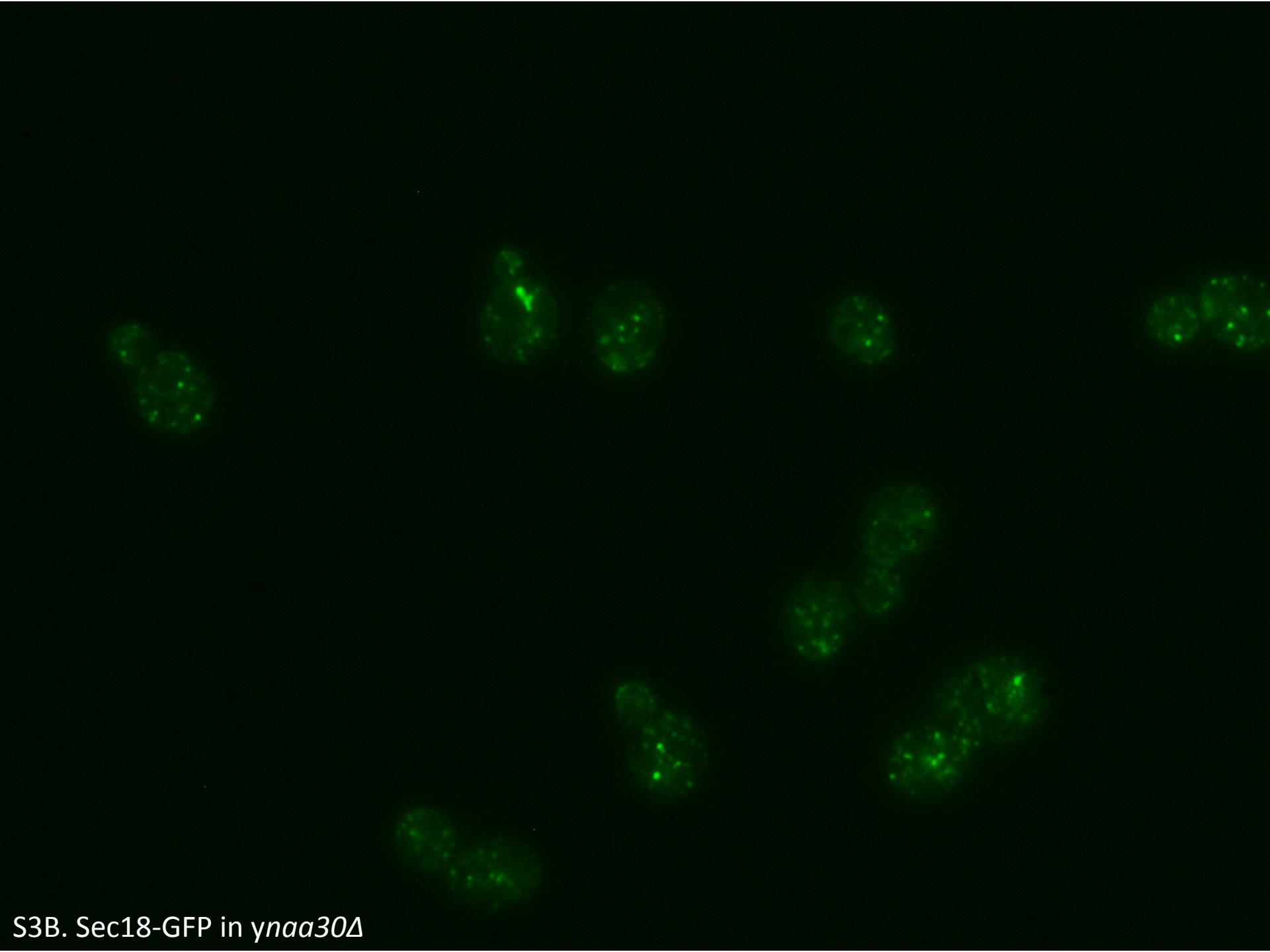

S3B. Sec18-GFP in *yna30Δ*

Supplement: Figure S3 — Full-field view of Sec18-GFP cells. The early Golgi localization of Sec18 in wild type cells (A) was maintained in naa30Δ cells (B). (PDF) [file pone.0061012.s003.pdf]

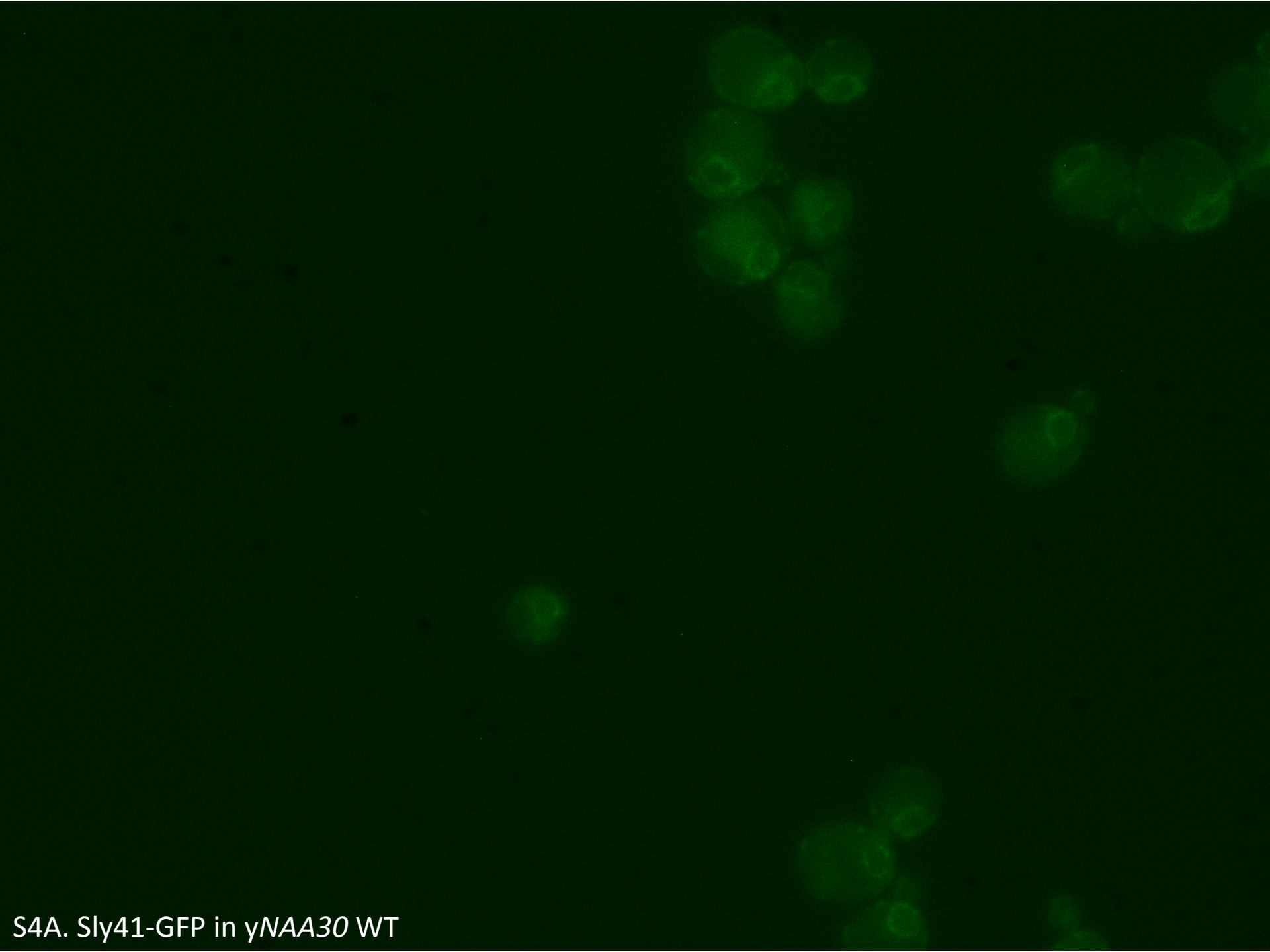

S4A. Sly41-GFP in yNAA30 WT

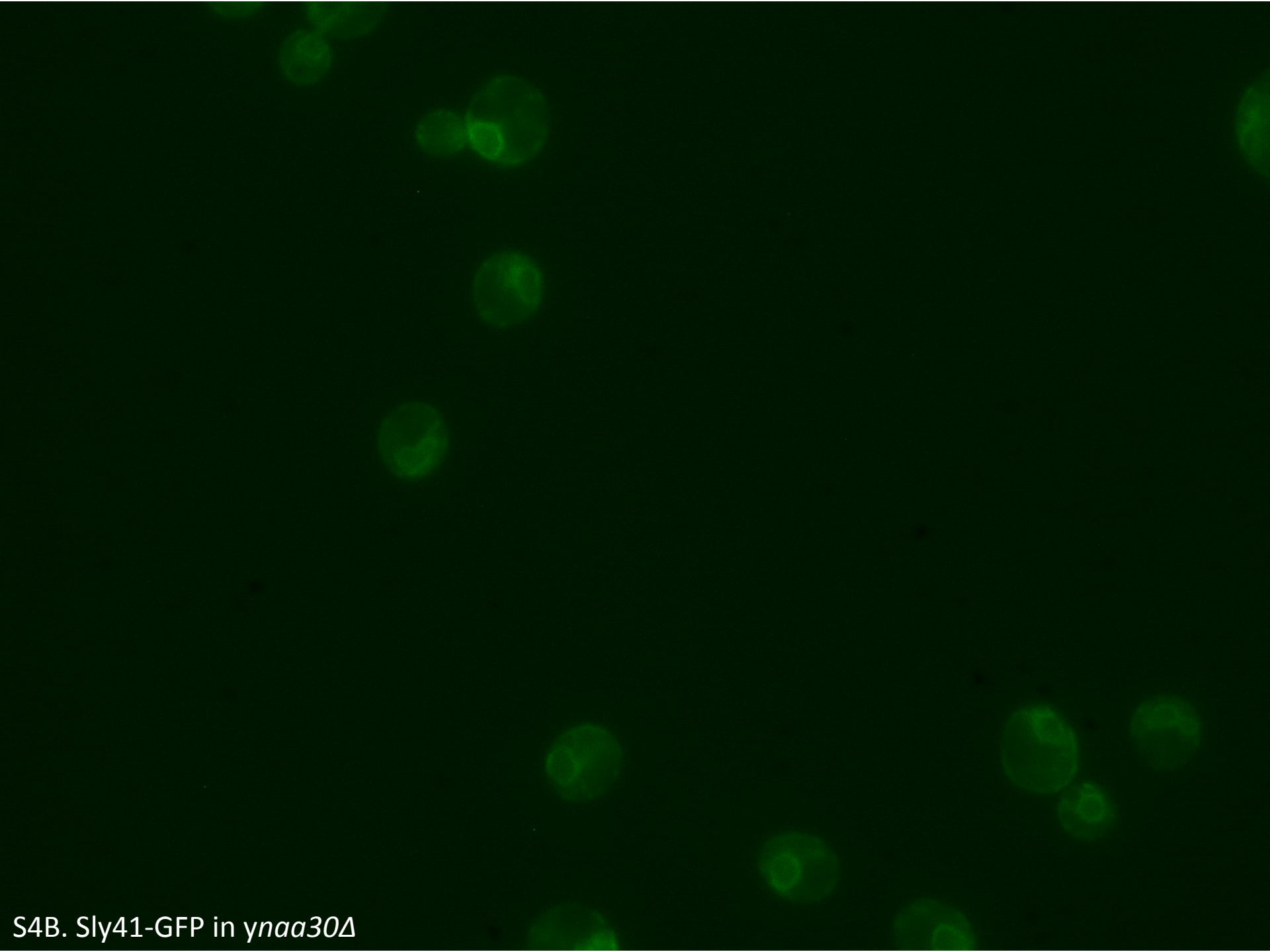

S4B. Sly41-GFP in *yna30Δ*

Supplement: Figure S4 — Full-field view of Sly41-GFP cells. The ER localization of Sly41 in wild type cells (A) was maintained in naa30Δ cells (B). (PDF) [file pone.0061012.s004.pdf]

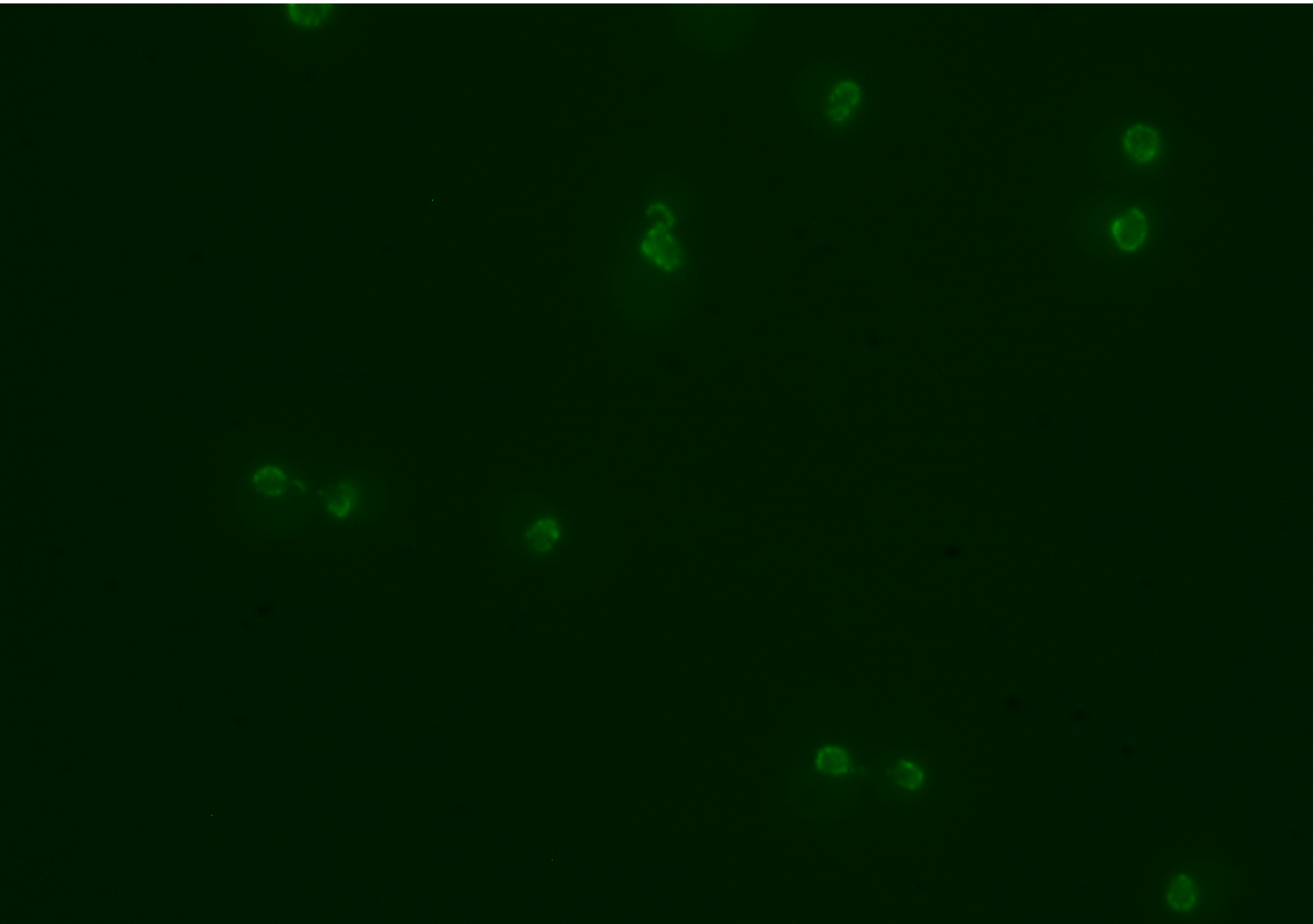

S5A. Nup157-GFP in yNAA30 WT

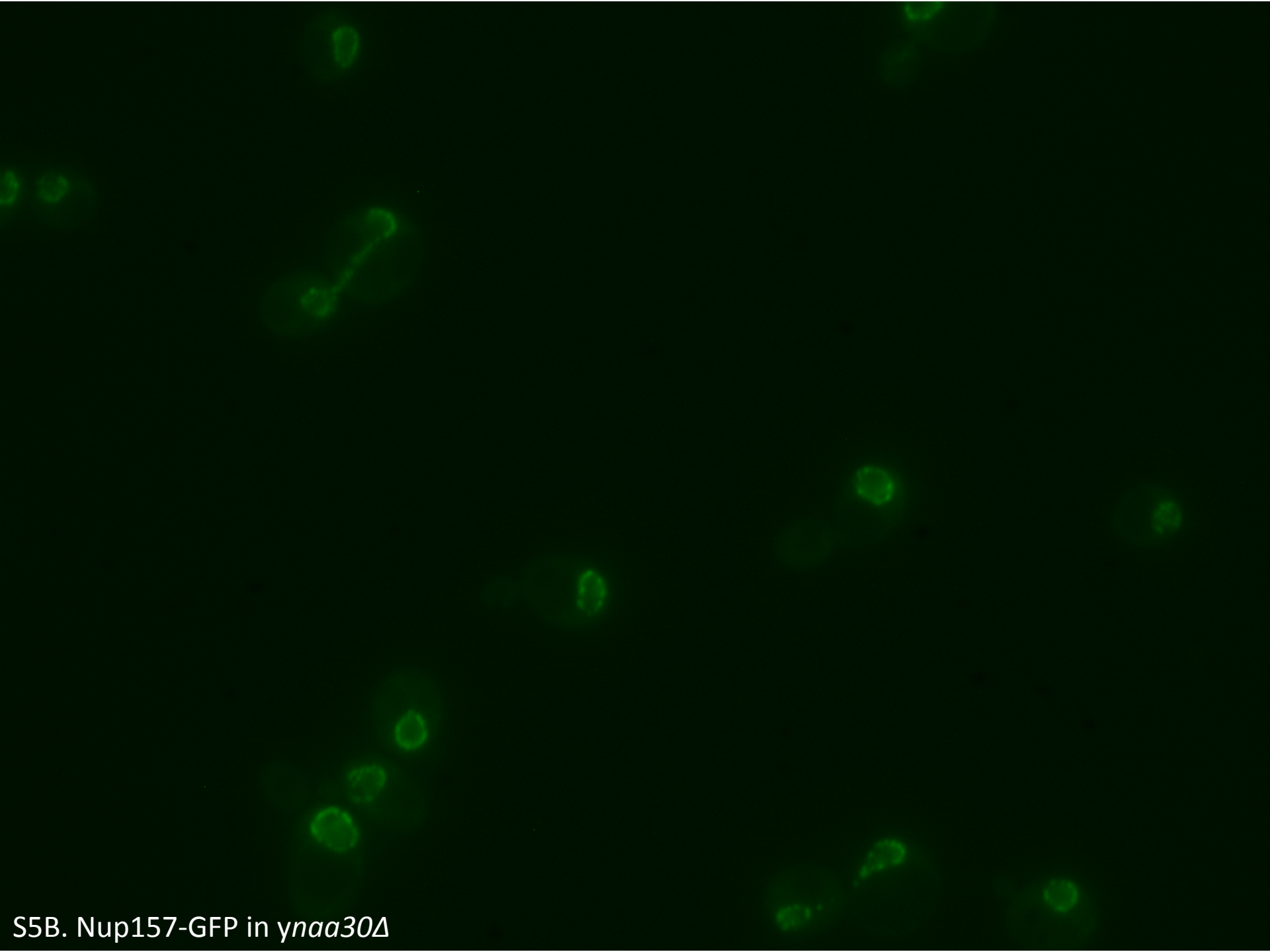

S5B. Nup157-GFP in *yna30Δ*

Supplement: Figure S5 — Full-field view of Nup157-GFP cells. The nuclear pore localization of Nup157 in wild type cells (A) was maintained in naa30Δ cells (B). (PDF) [file pone.0061012.s005.pdf]

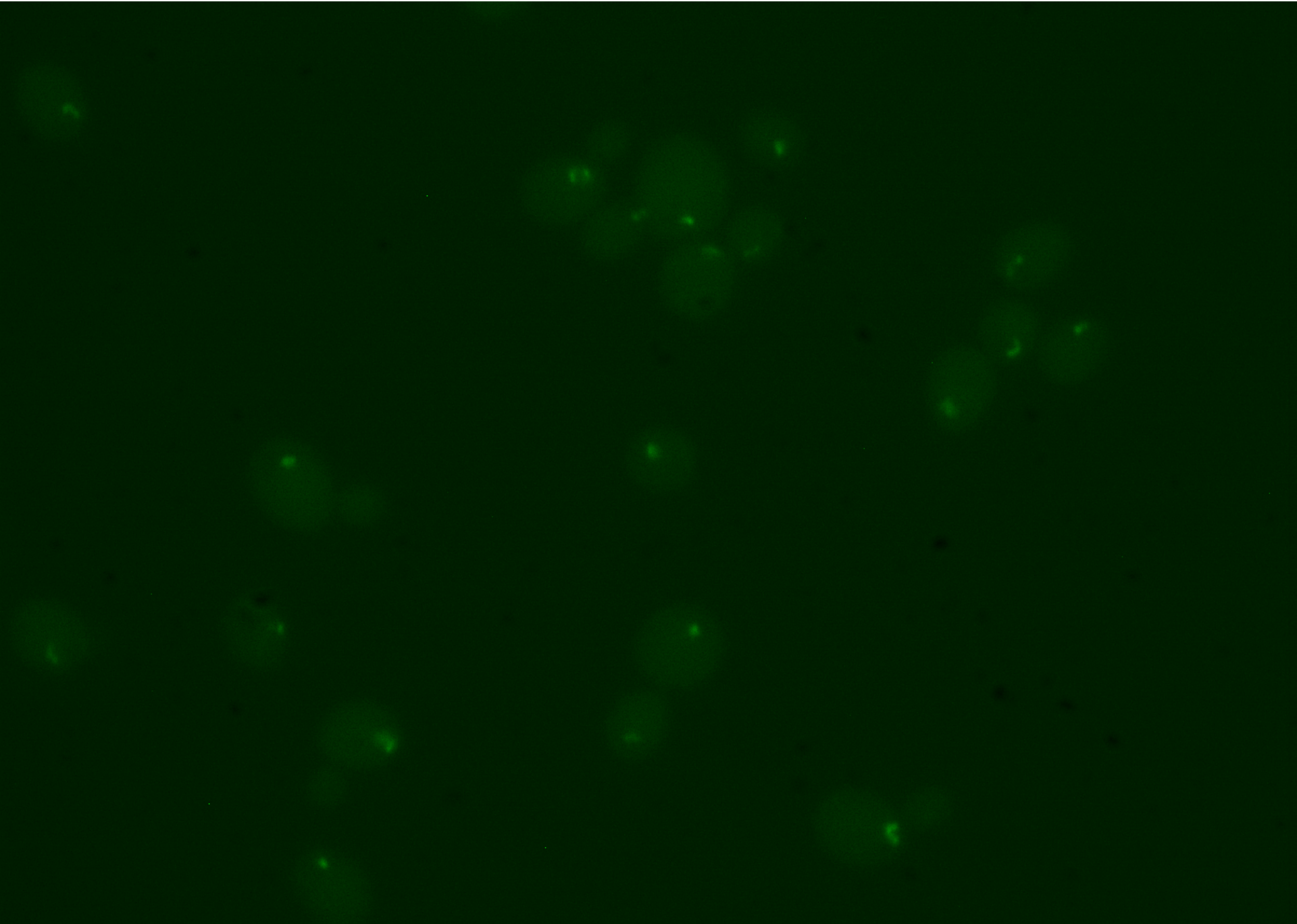

S6A. Rrn11-GFP in yNAA30 WT

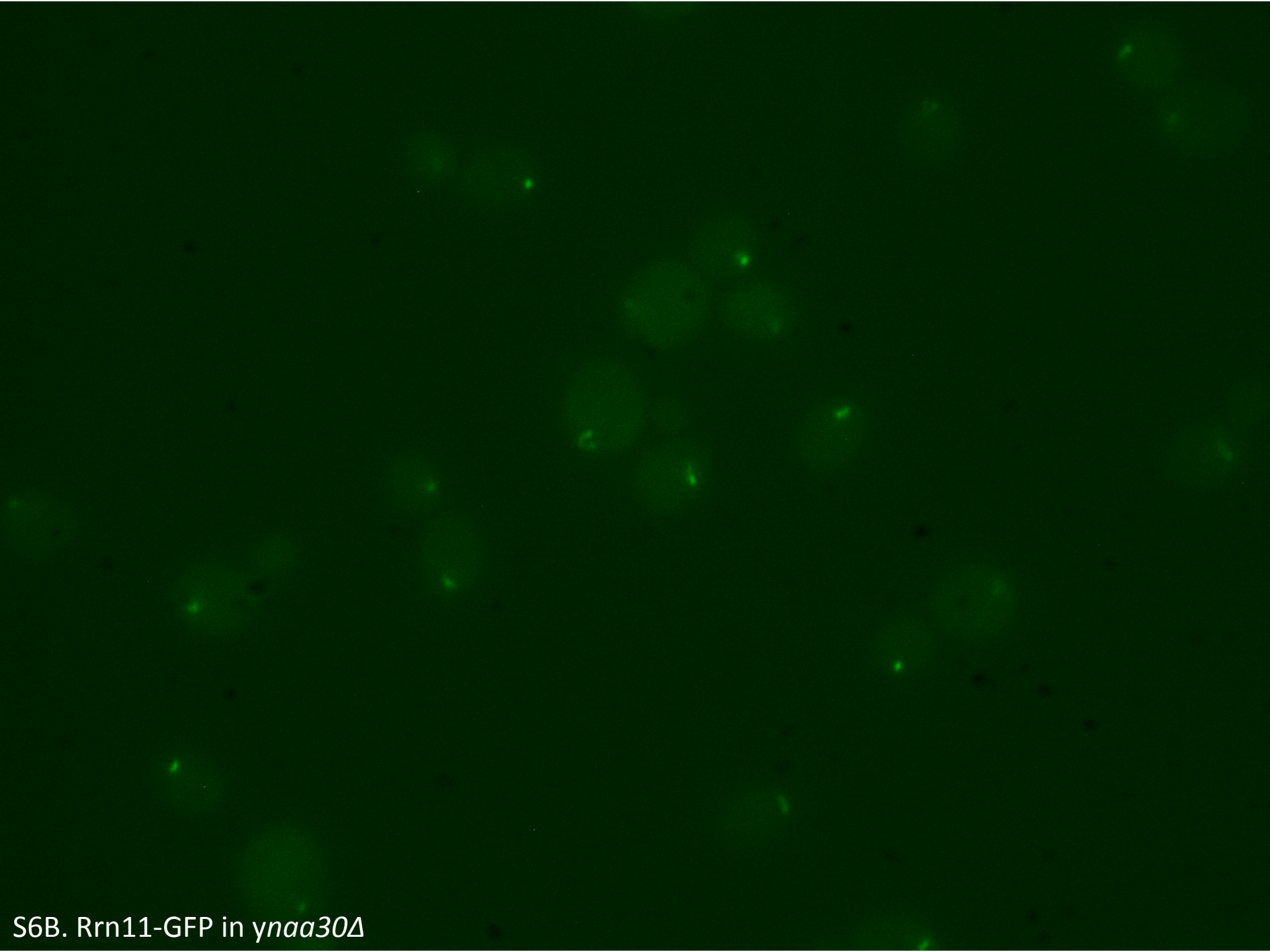

S6B. Rrn11-GFP in *yna30Δ*

Supplement: Figure S6 — Full-field view of Rrn11-GFP cells. The nucleolus localization of Rrn11 in wild type cells (A) was maintained in naa30Δ cells (B). (PDF) [file pone.0061012.s006.pdf]

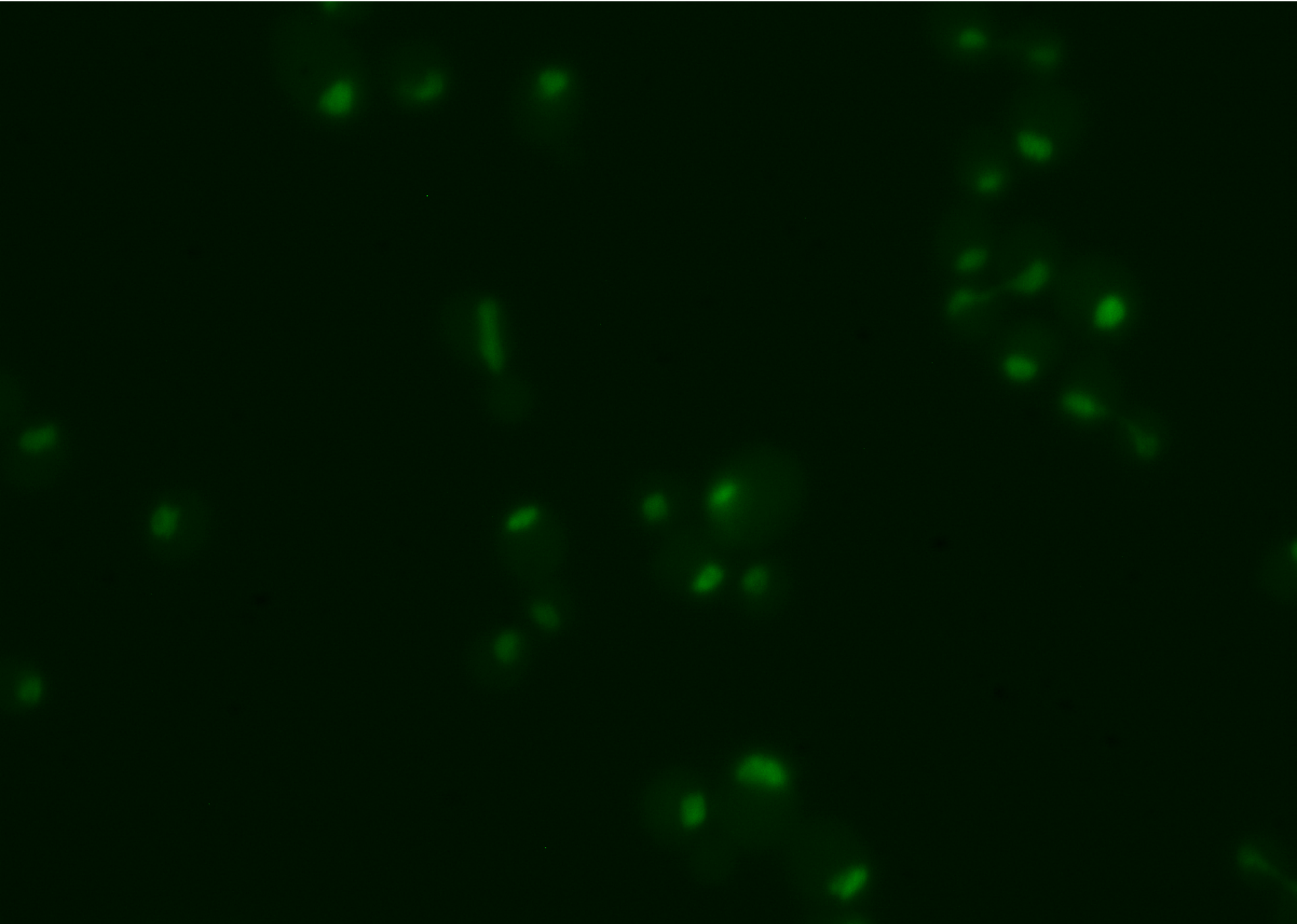

S7A. Rfc2-GFP in *yNAA30* WT

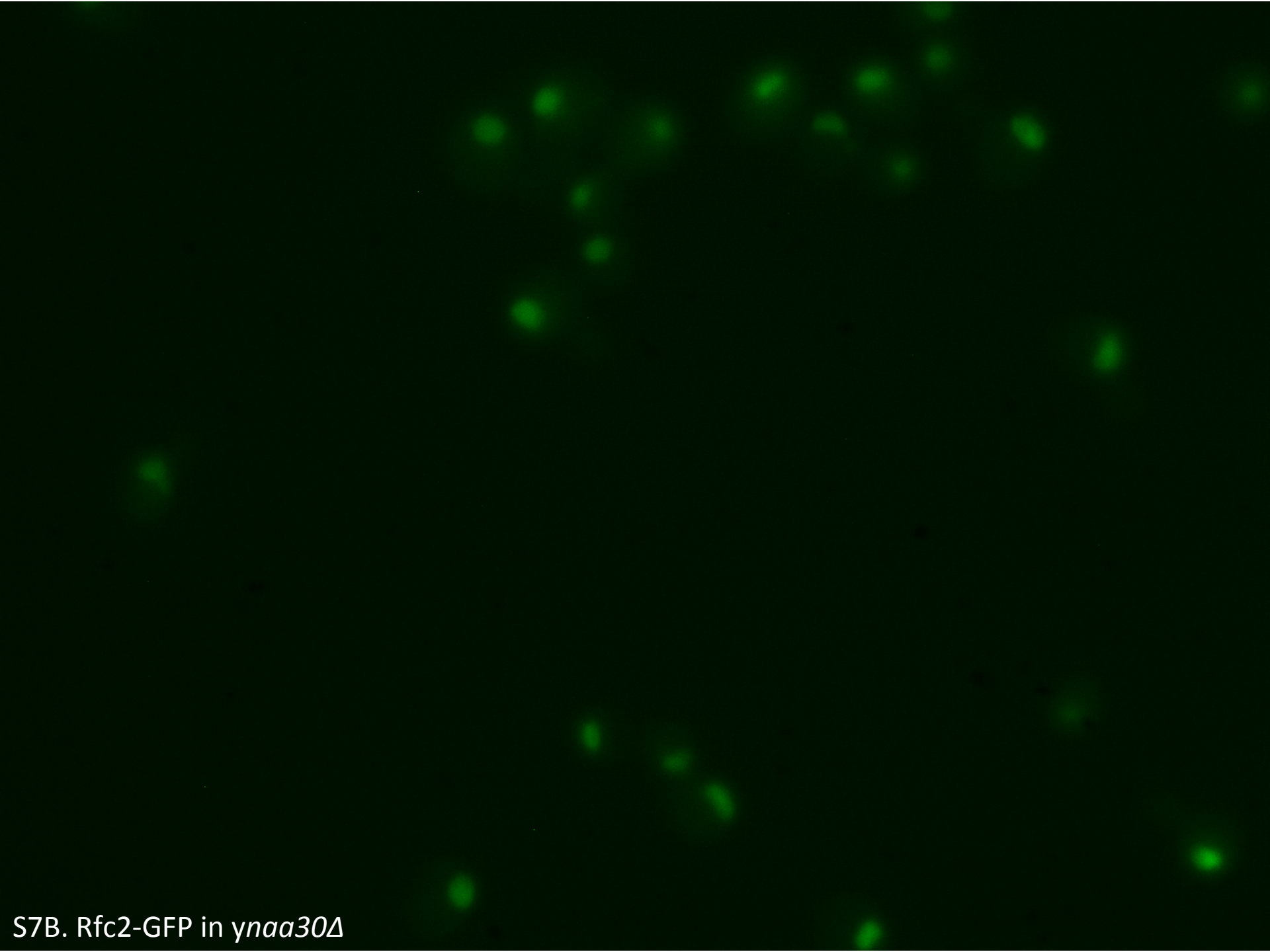

S7B. Rfc2-GFP in *yna30Δ*

Supplement: Figure S7 — Full-field view of Rfc2-GFP cells. The nuclear localization of Rfc2 in wild type cells (A) was maintained in naa30Δ cells (B). (PDF) [file pone.0061012.s007.pdf]

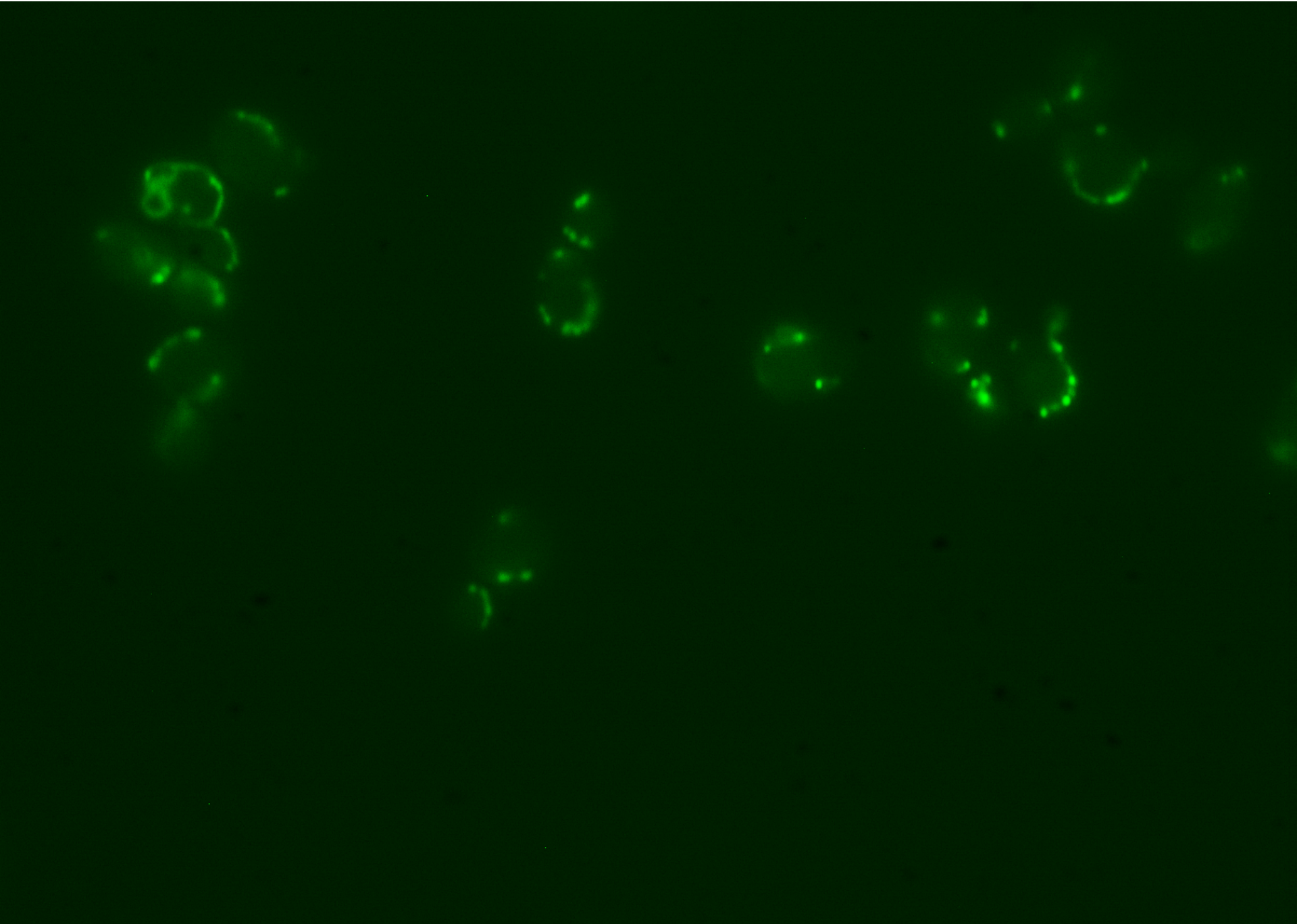

S8A. Ymr31-GFP in yNAA30 WT

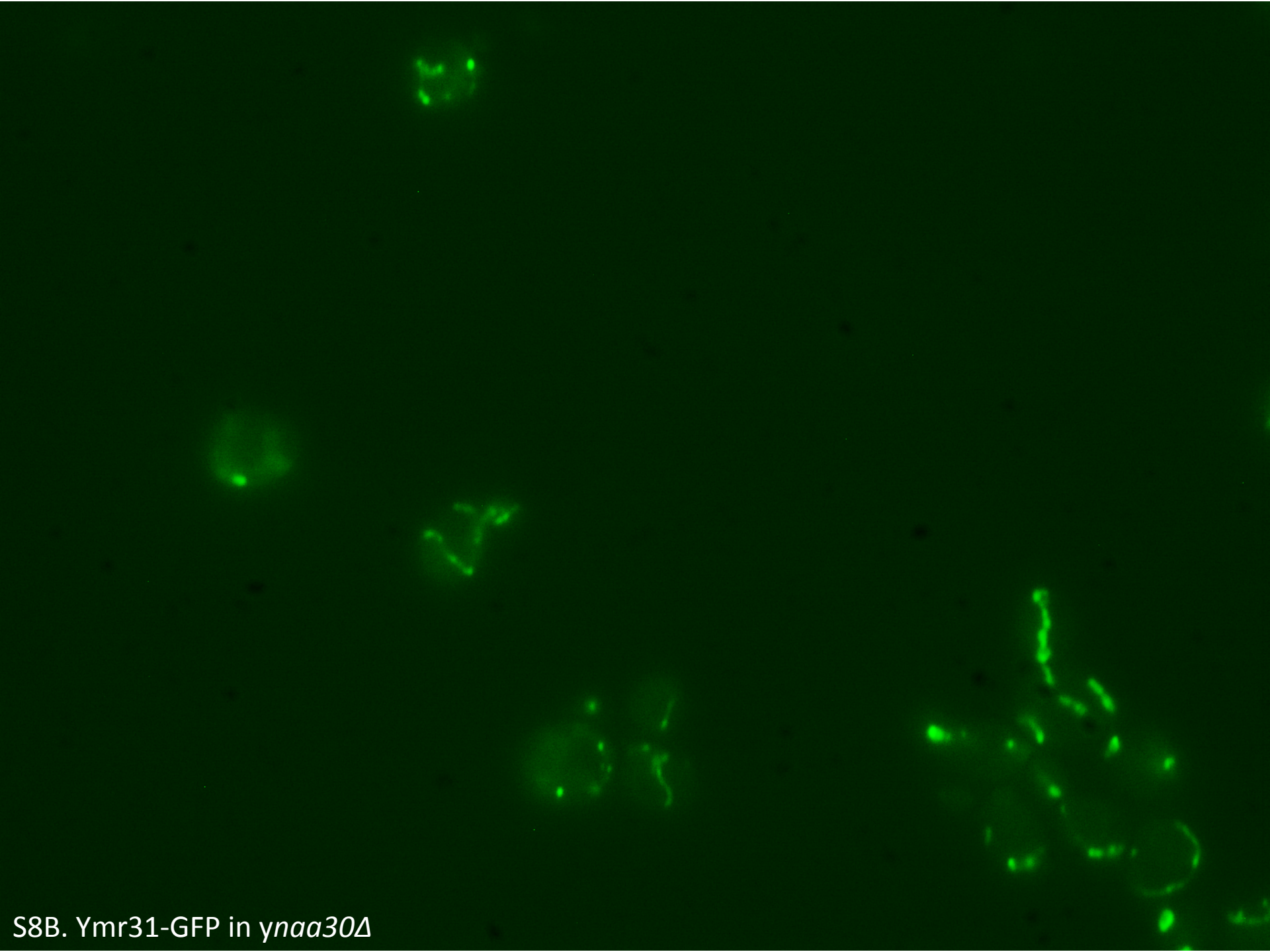

S8B. Ymr31-GFP in *yna30Δ*

Supplement: Figure S8 — Full-field view of Ymr31-GFP cells. The mitochondrial localization of Ymr31 in wild type cells (A) was maintained in naa30Δ cells (B). (PDF) [file pone.0061012.s008.pdf]

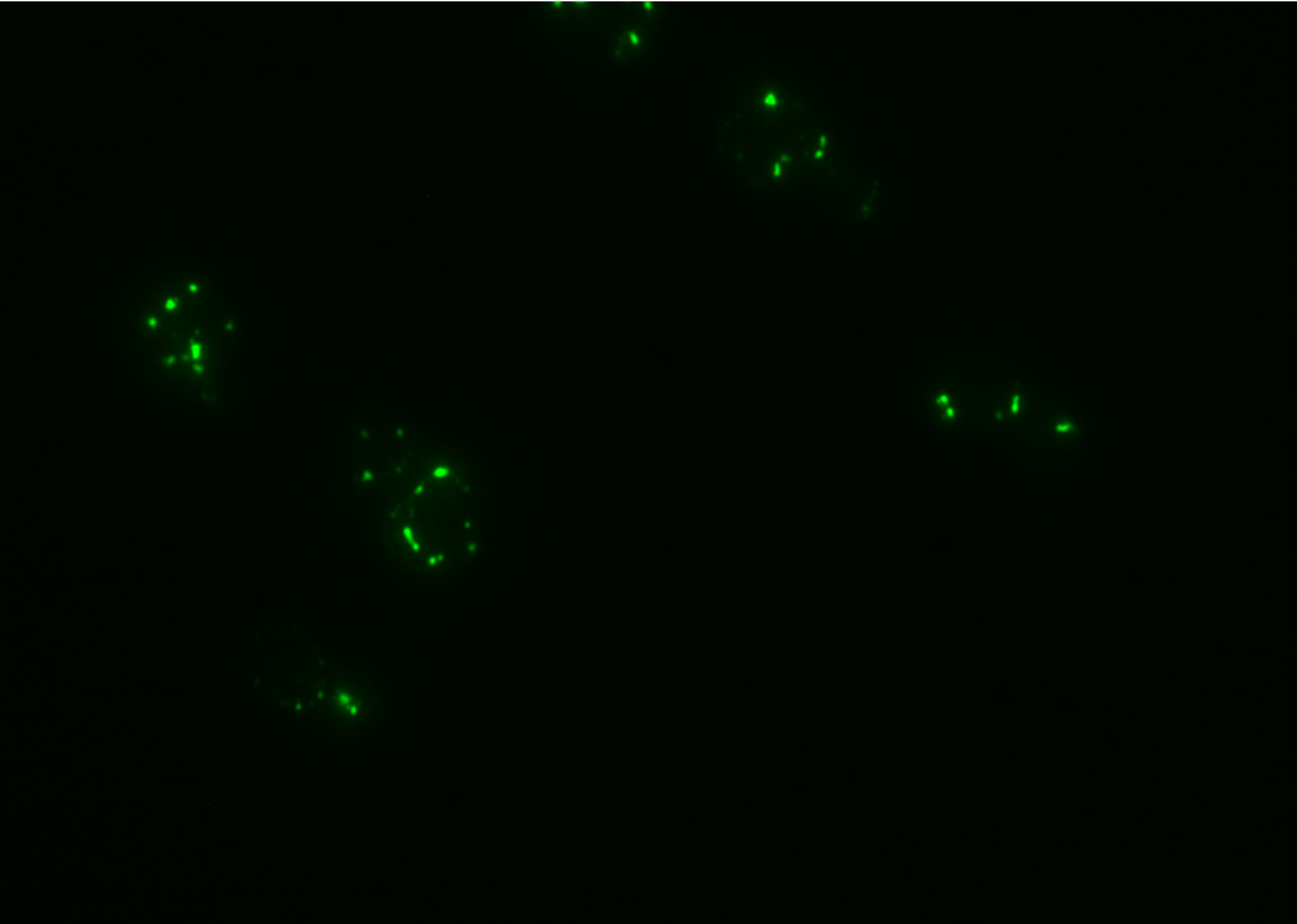

S9A. Pda1-GFP in yNAA30 WT

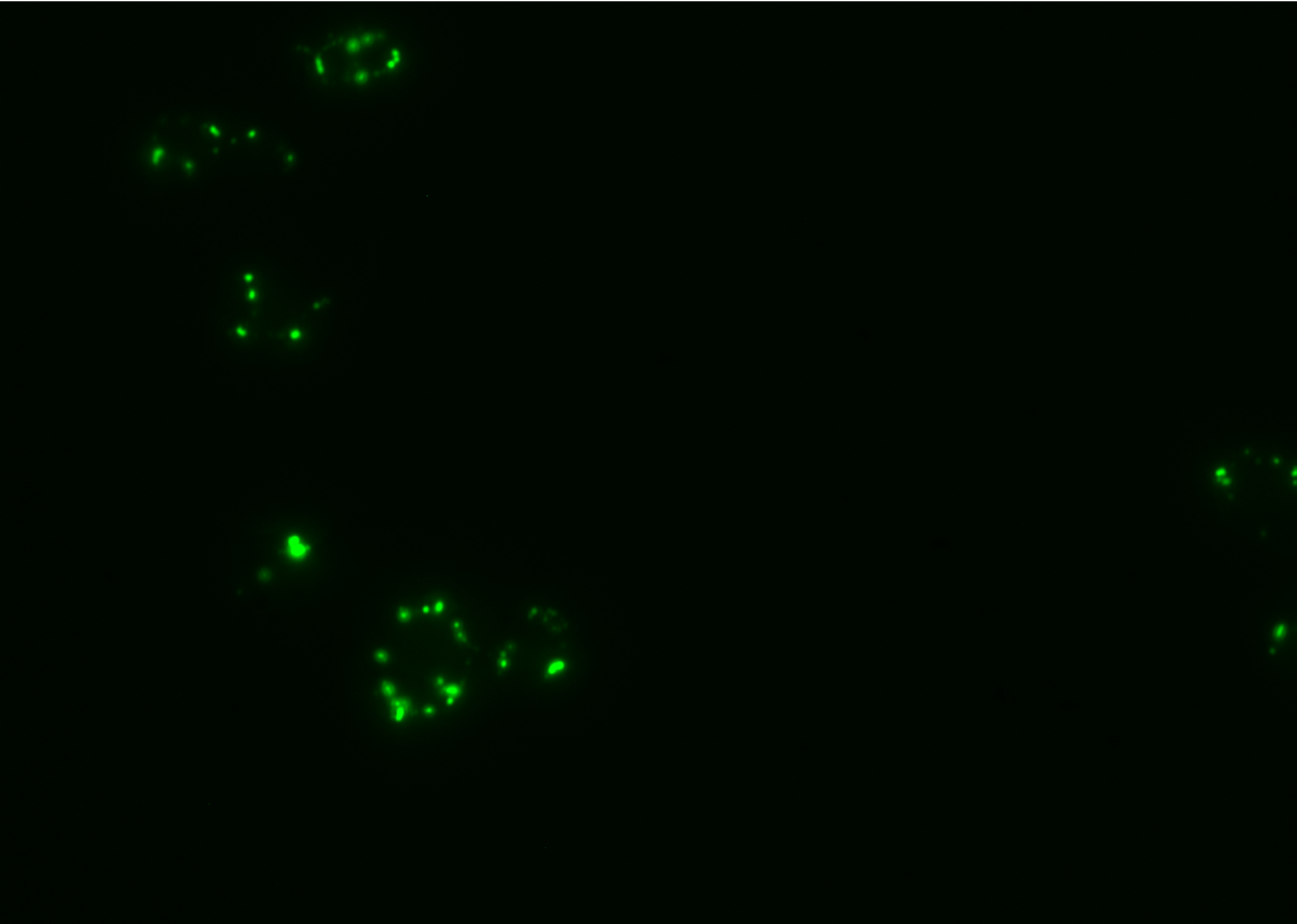

S9B. Pda1-GFP in *yna30Δ*

Supplement: Figure S9 — Full-field view of Pda1-GFP cells. The mitochondrial localization of Pda1 in wild type cells (A) was maintained in naa30Δ cells (B). (PDF) [file pone.0061012.s009.pdf]

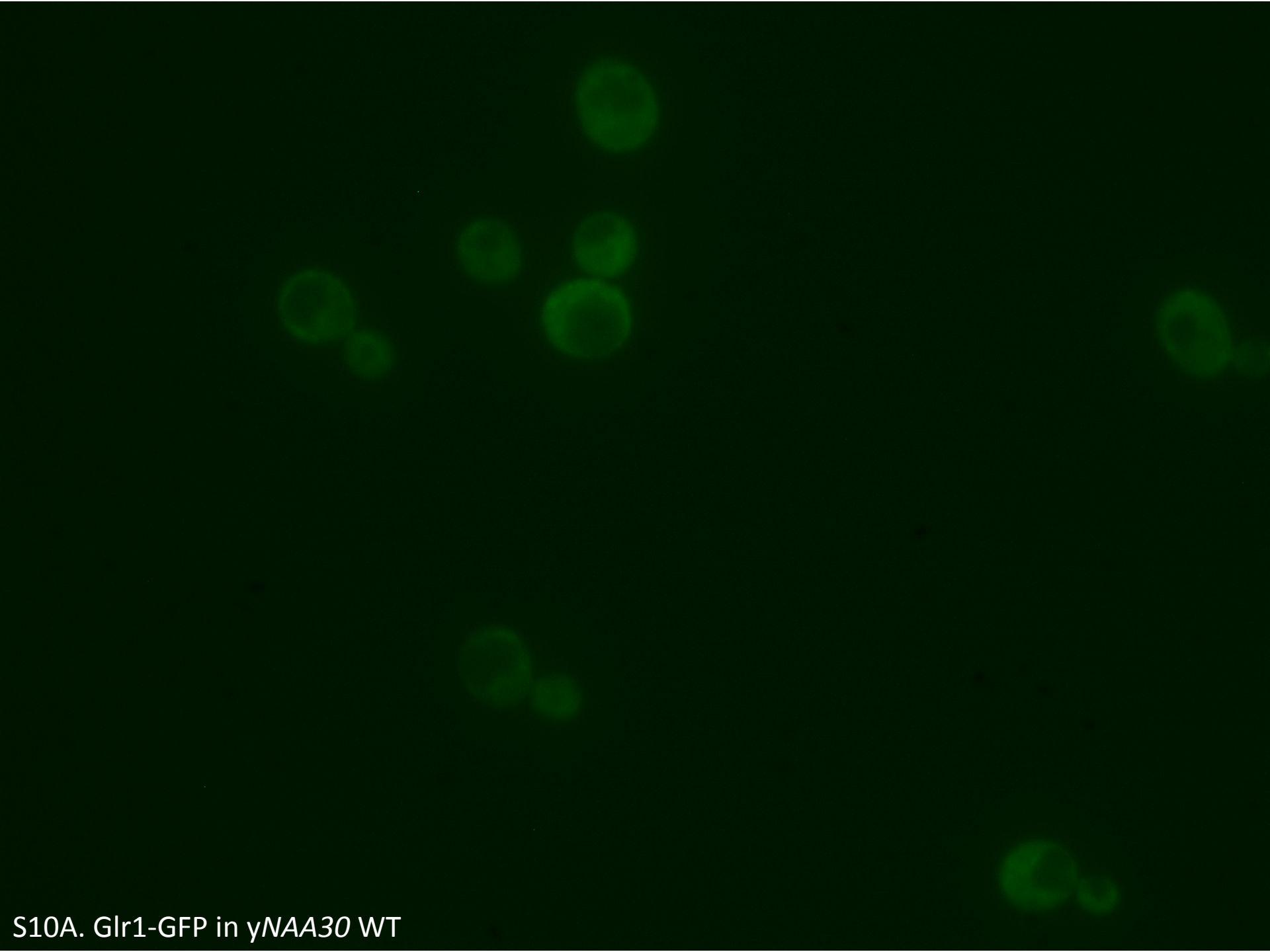

S10A. Glr1-GFP in yNAA30 WT

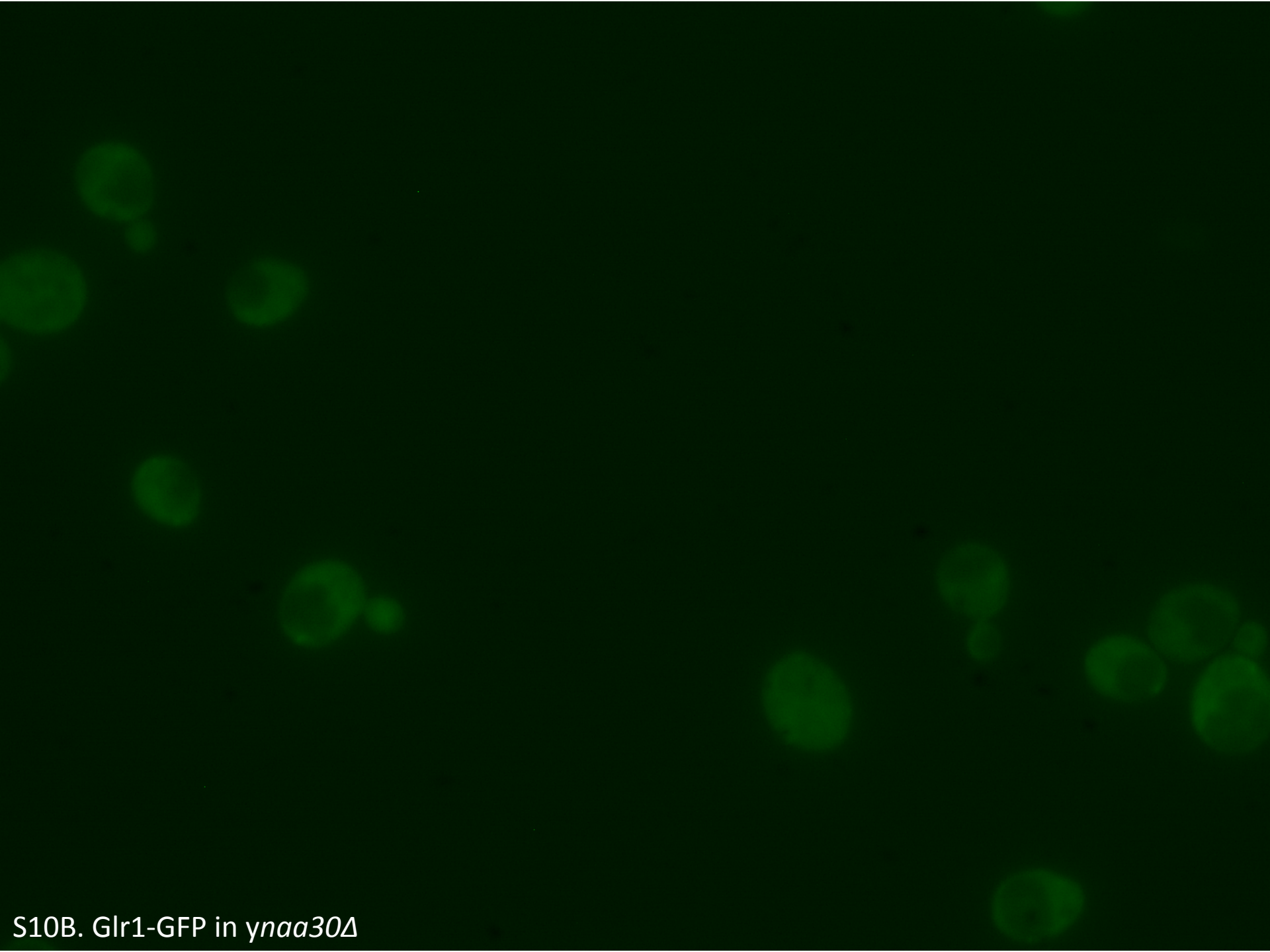

S10B. Glr1-GFP in *yna30Δ*

Supplement: Figure S10 — Full-field view of Glr1-GFP cells. The cytosolic and nuclear localization of Glr1 in wild type cells (A) was maintained in naa30Δ cells (B). The described mitochondrial localization could not be distinguished in either wild type or naa30Δ cells. (PDF) [file pone.0061012.s010.pdf]

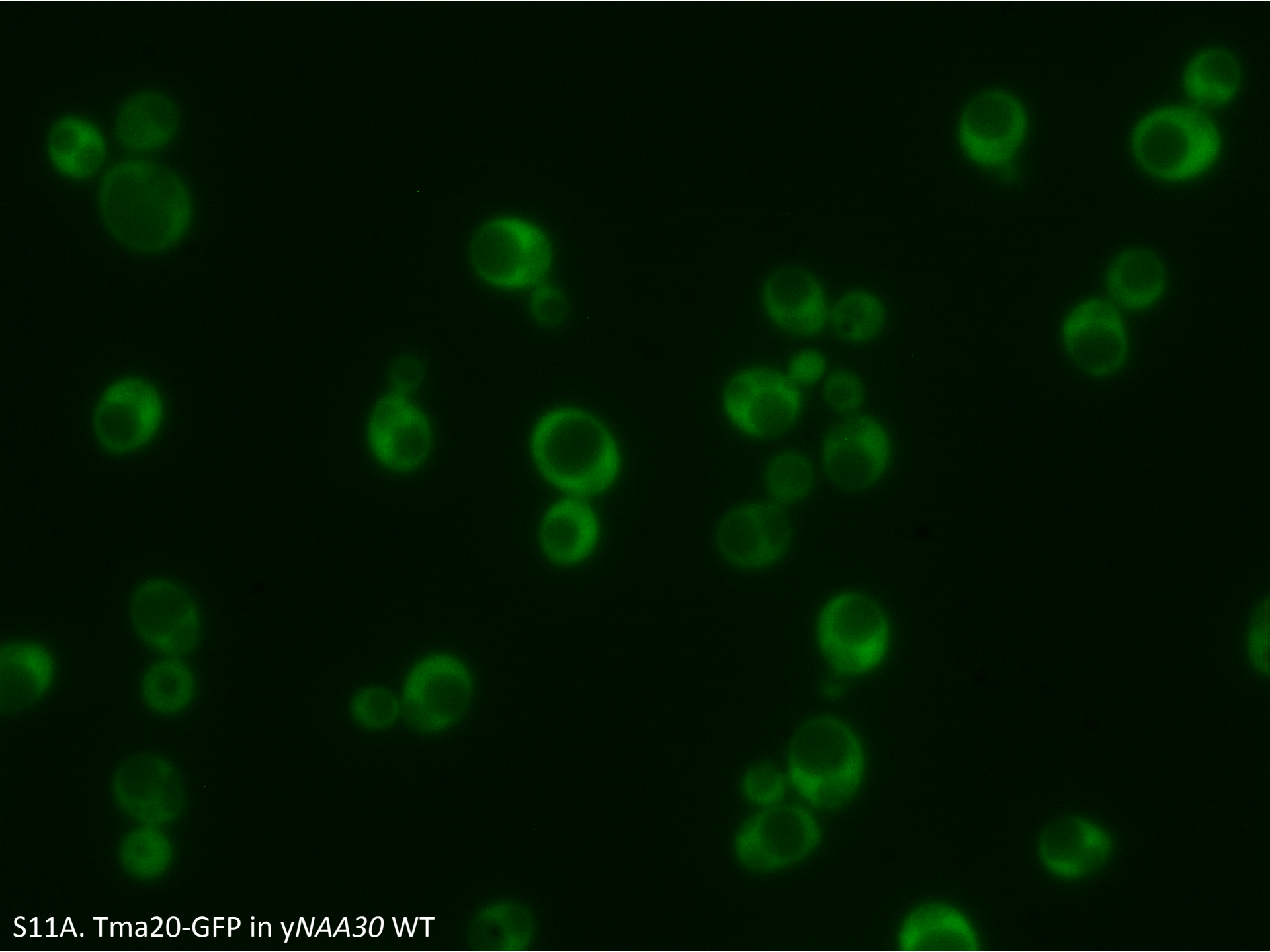

S11A. Tma20-GFP in yNAA30 WT

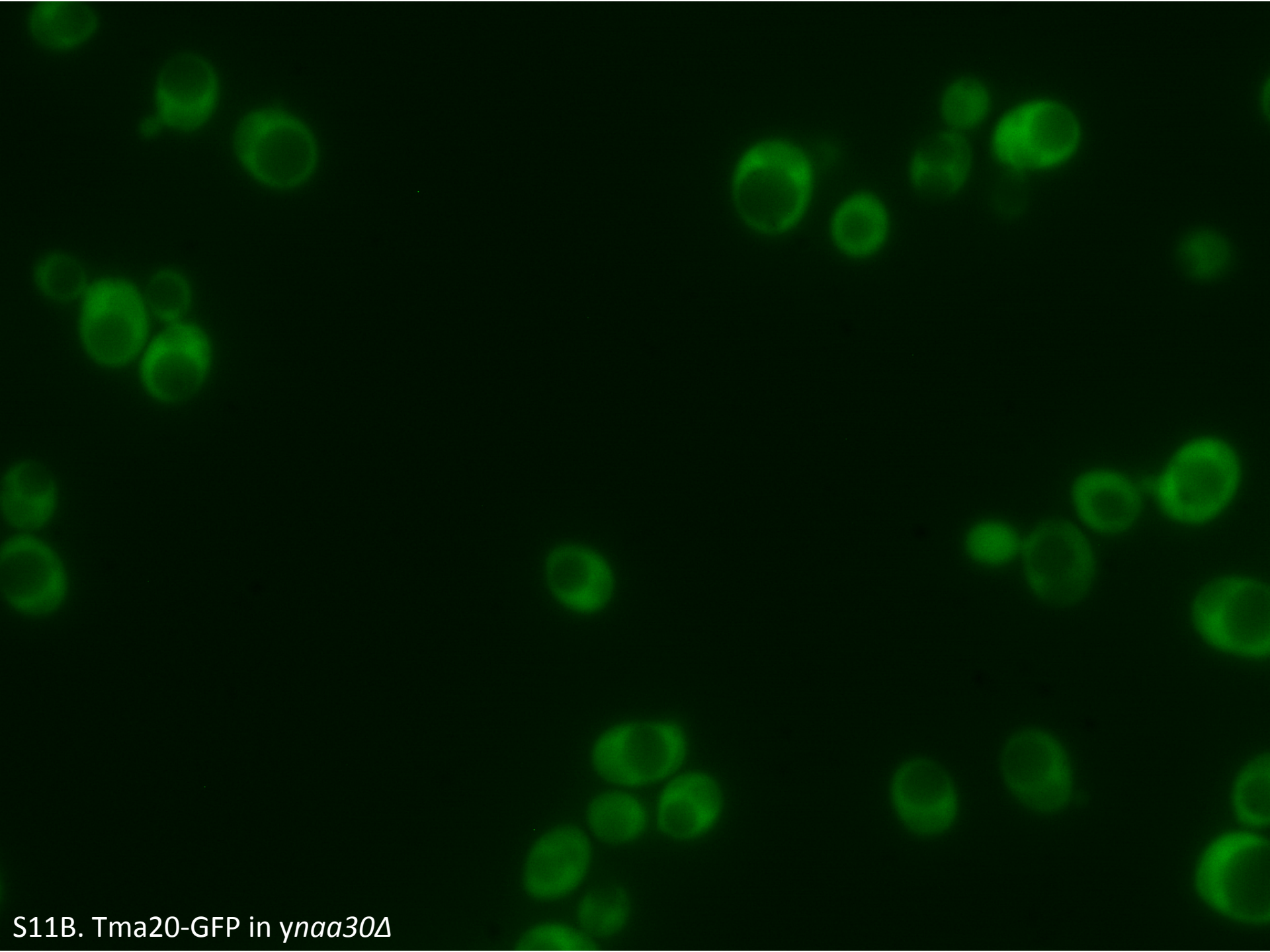

S11B. Tma20-GFP in *yna30Δ*

Supplement: Figure S11 — Full-field view of Tma20-GFP cells. The cytosolic localization of Tma20 in wild type cells (A) was maintained in naa30Δ cells (B). (PDF) [file pone.0061012.s011.pdf]

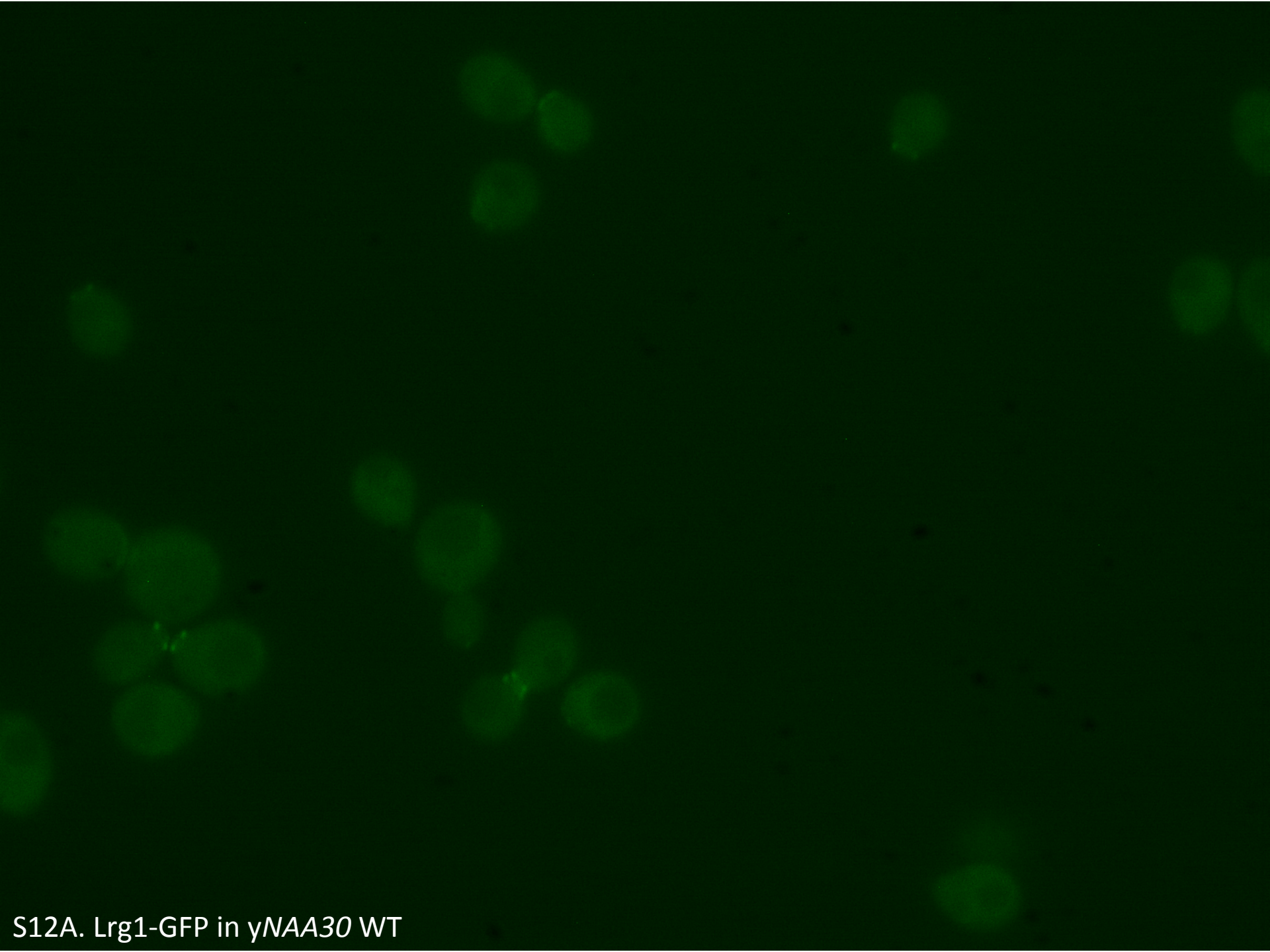

S12A. Lrg1-GFP in yNAA30 WT

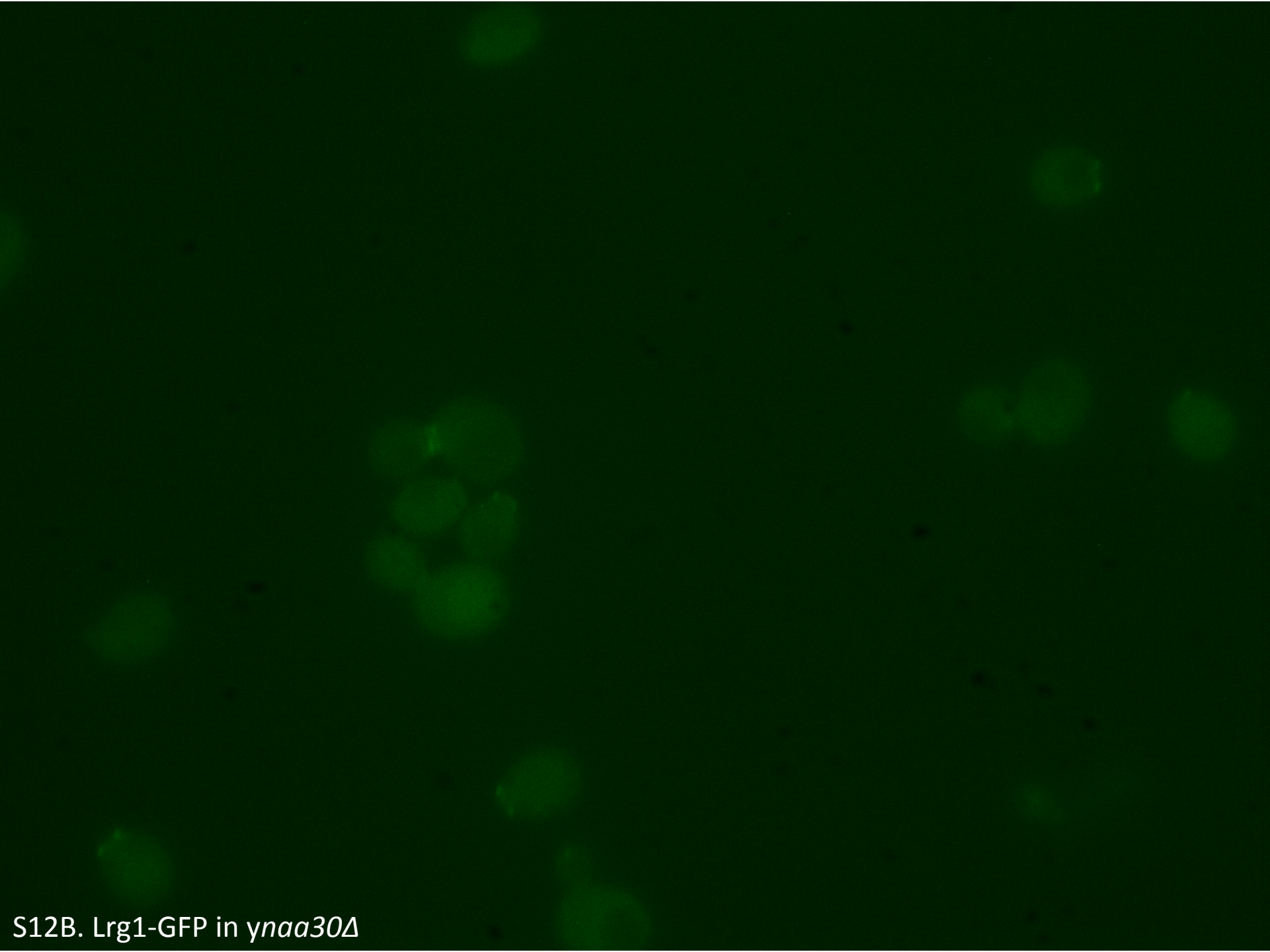

S12B. Lrg1-GFP in *yna30Δ*

Supplement: Figure S12 — Full-field view of Lrg1-GFP cells. The bud-neck localization of Lrg1 in wild type cells (A) was maintained in naa30Δ cells (B). (PDF) [file pone.0061012.s012.pdf]

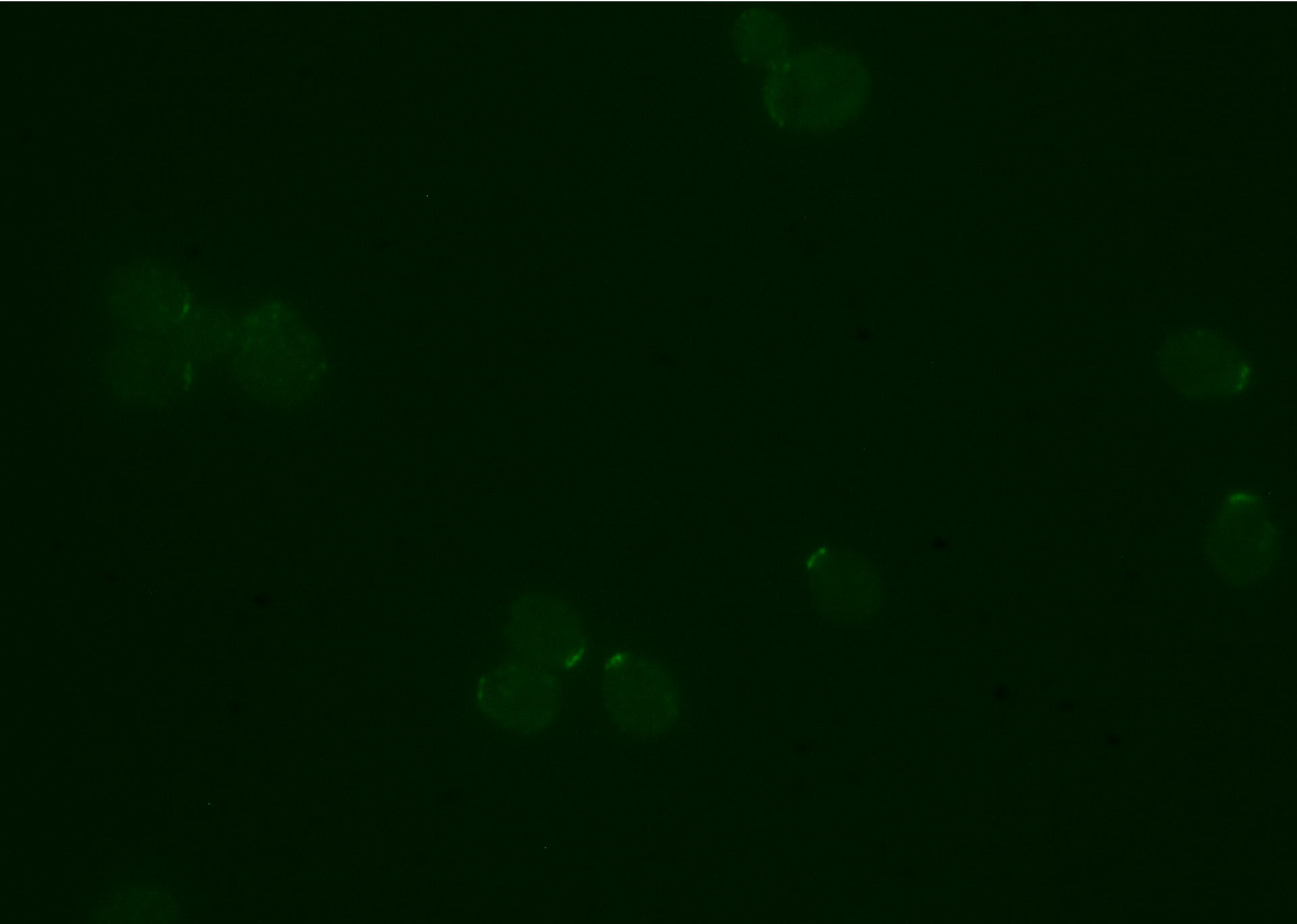

S13A. Bem1-GFP in yNAA30 WT

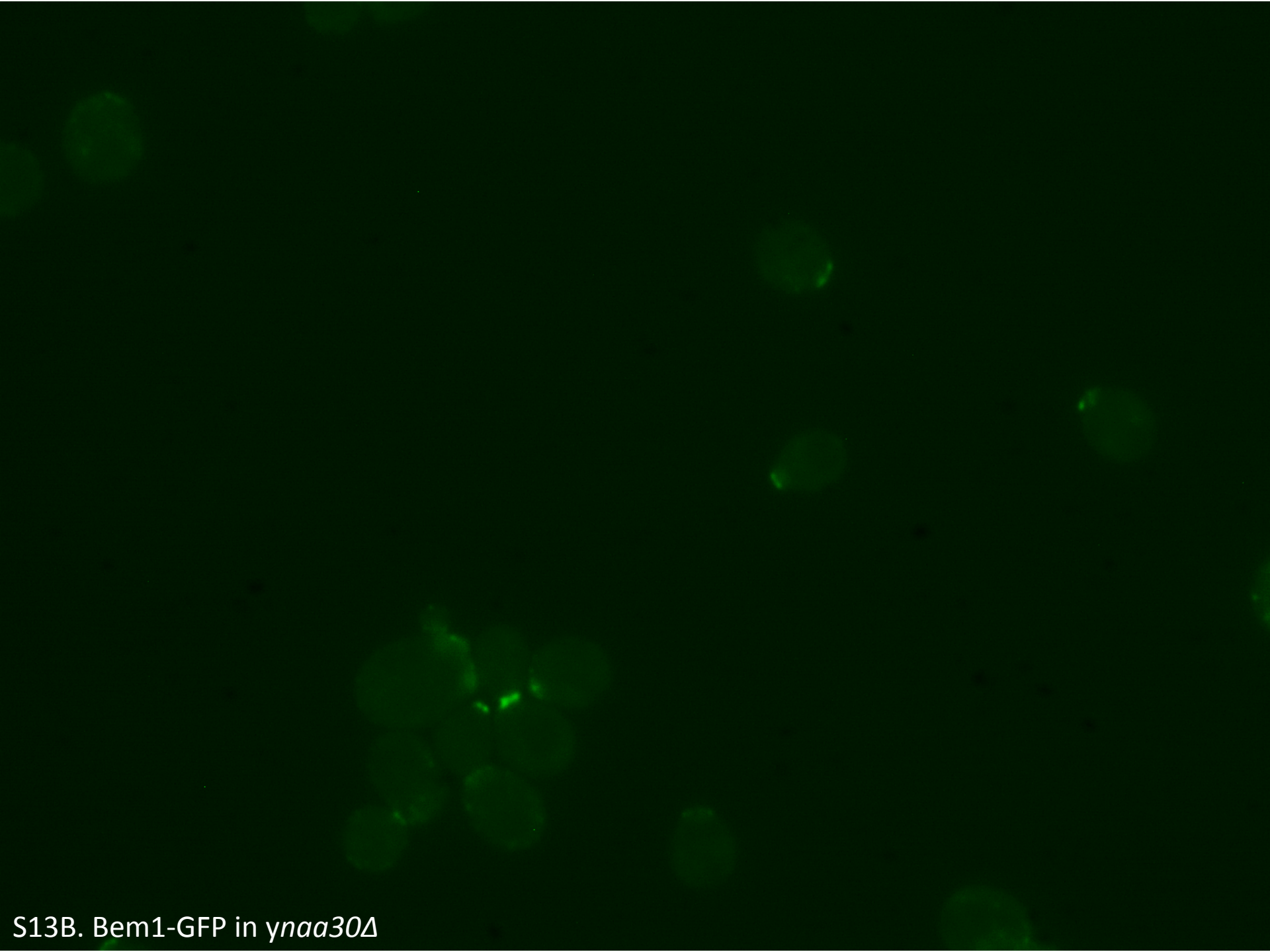

S13B. Bem1-GFP in *yna30Δ*

Supplement: Figure S13 — Full-field view of Bem1-GFP cells. The bud-neck localization of Bem1 in wild type cells (A) was maintained in naa30Δ cells (B). (PDF) [file pone.0061012.s013.pdf]

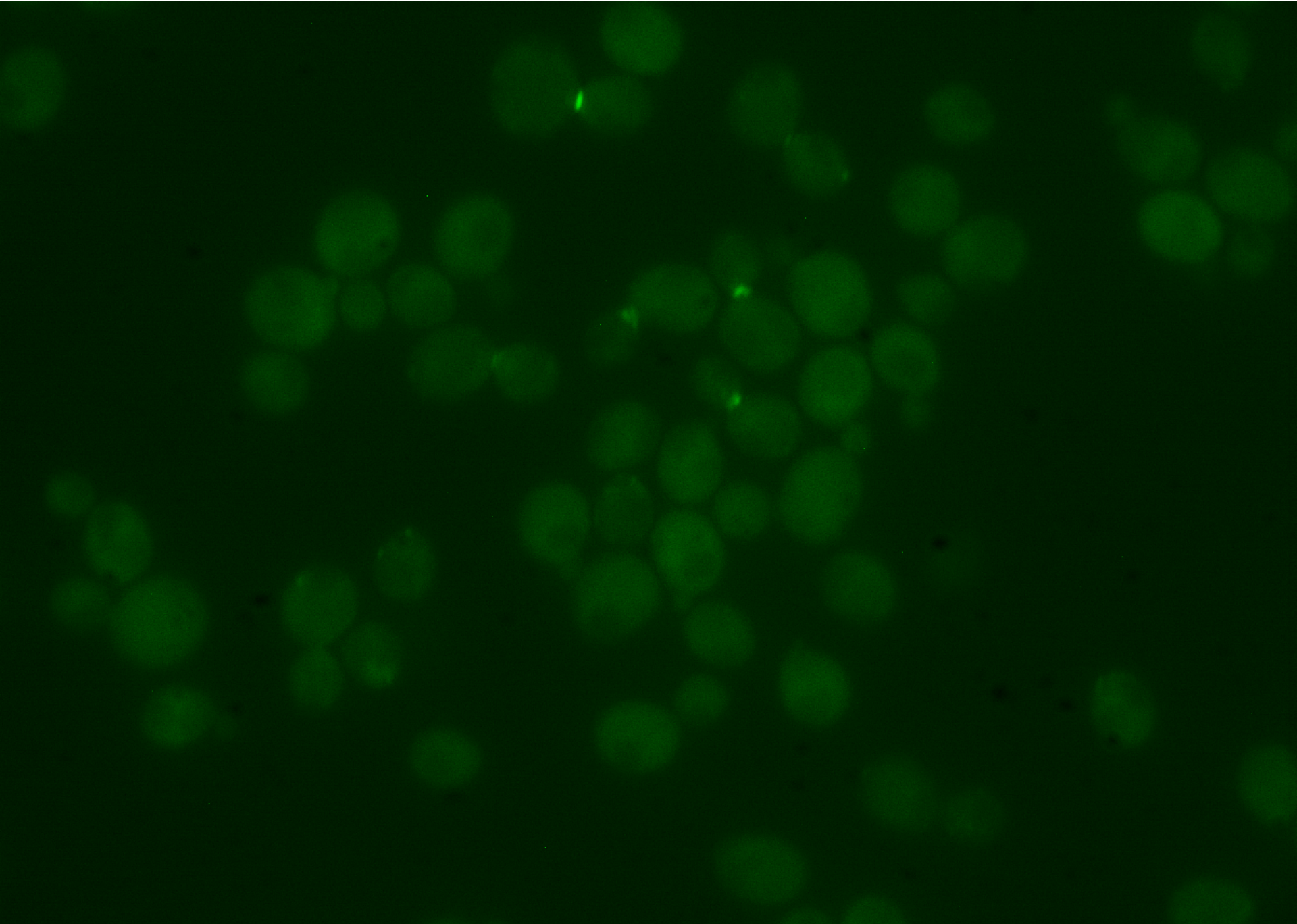

S14A. PxI1-GFP in yNAA30 WT

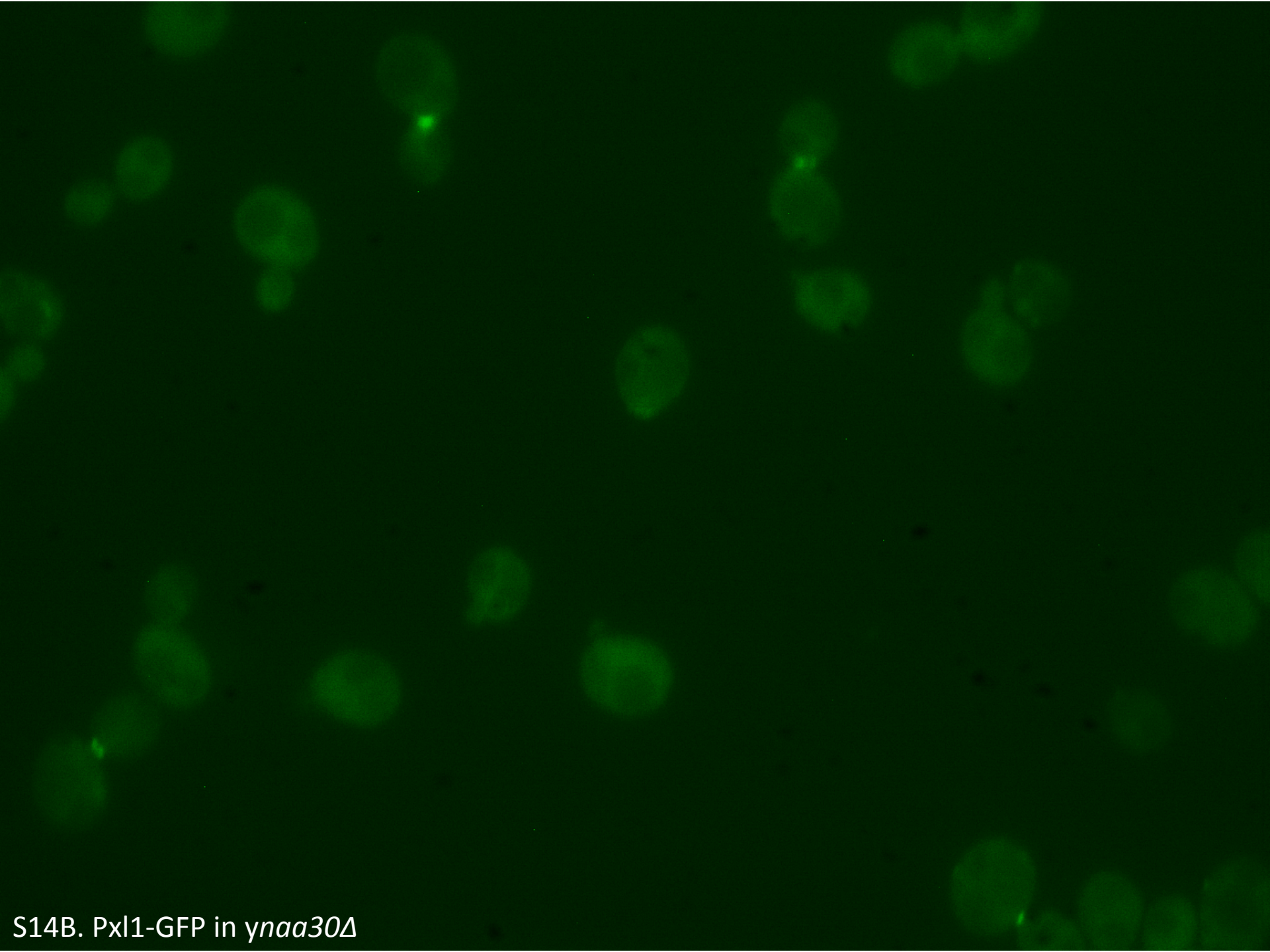

S14B. Pxl1-GFP in *yna* $\alpha$ 30 $\Delta$

Supplement: Figure S14 — Full-field view of Pxl1-GFP cells. The localization of Pxl1 to sites of polarized growth in wild type cells (A) was maintained in naa30Δ cells (B). (PDF) [file pone.0061012.s014.pdf]

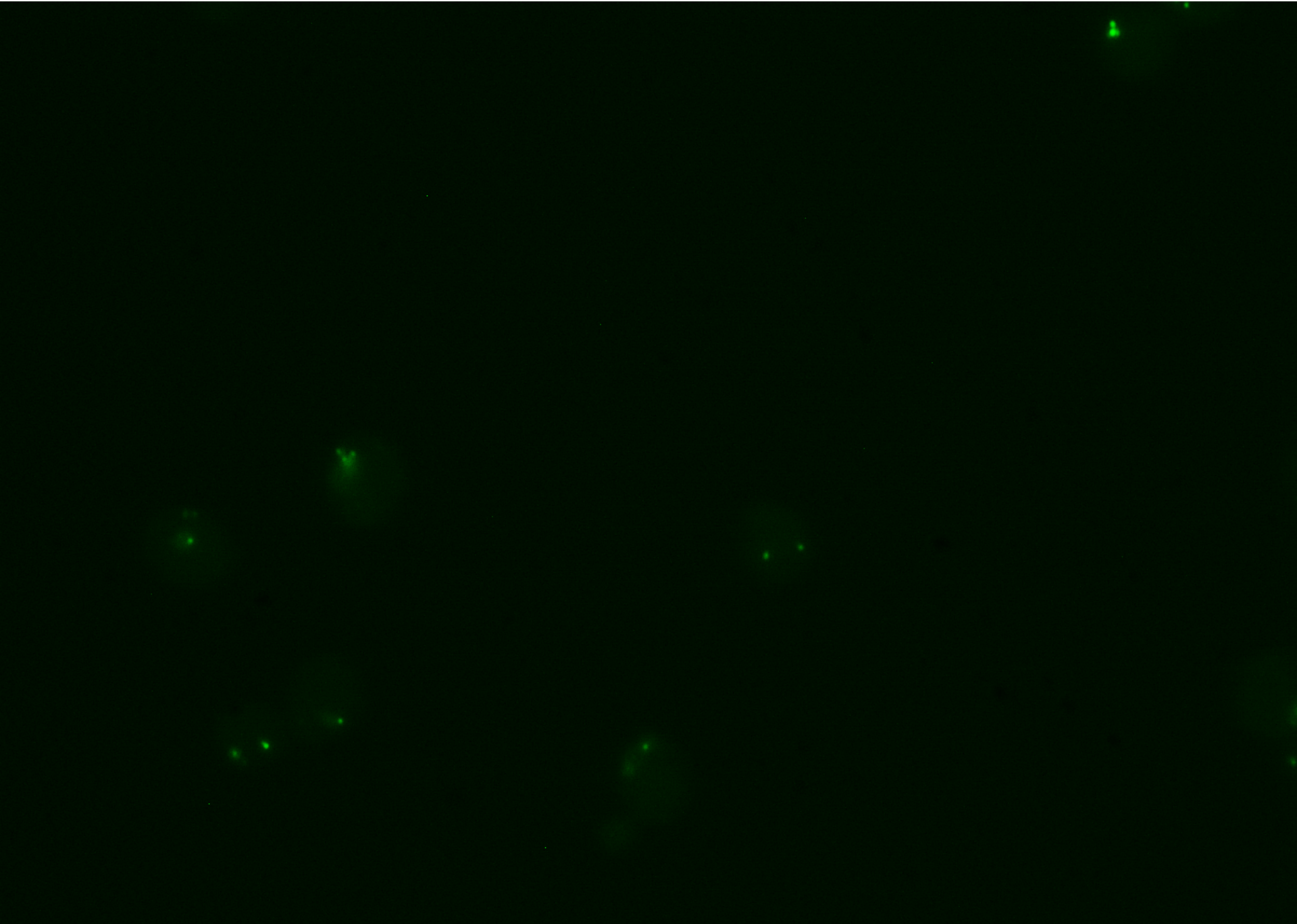

S15A. Tgl1-GFP in yNAA30 WT

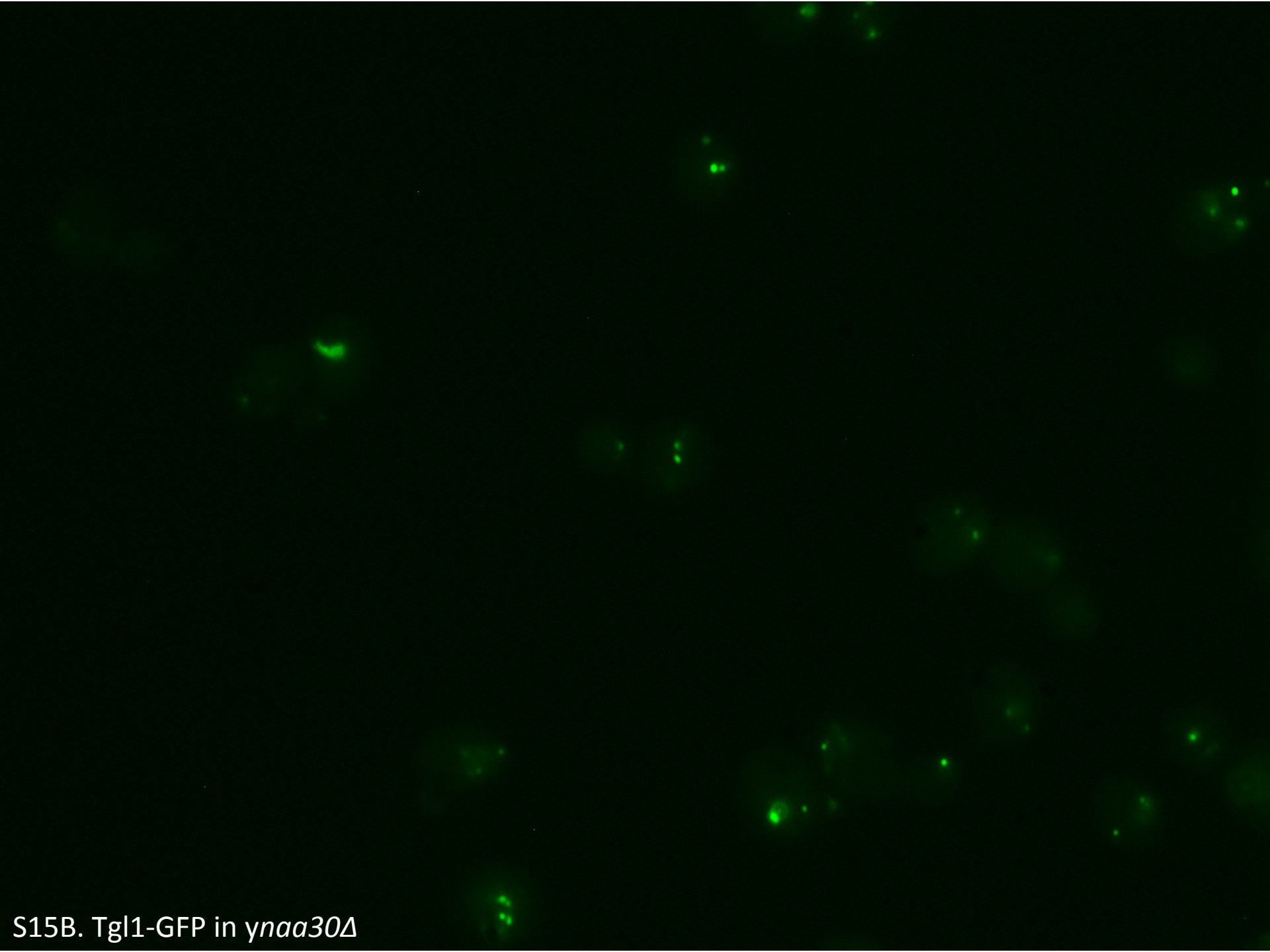

S15B. Tg1-GFP in *yna30Δ*

Supplement: Figure S15 — Full-field view of Tgl1-GFP cells. The lipid particle localization of Tgl1 in wild type cells (A) was maintained in naa30Δ cells (B). (PDF) [file pone.0061012.s015.pdf]
