# Supplementary material for: N-Terminal Acetylation by NatC Is Not a General Determinant for Substrate Subcellular Localization in Saccharomyces cerevisiae
Source: PLoS One. 2013 Apr 15;8(4):e61012. doi: 10.1371/journal.pone.0061012 (PMC3626706; doi:10.1371/journal.pone.0061012)
Supplement: Table S1 — Yeast strains used in this study. (PDF) [file pone.0061012.s016.pdf]

Table S1. Yeast strains used in this study.

Strain	Genotype
TA36	BY4742 *Matα; his3Δ1; leu2Δ0; lys2Δ0; ura3Δ0*
TA44	BY4742; *Matα; his3Δ1; leu2Δ0; lys2Δ0; ura3Δ0; naa30Δ::kanMX4*
TA256	BY4741; *Mata; his3Δ1; leu2Δ0; met15Δ0; ura3Δ0; ARL3::GFP-HIS3MX6*
TA266	BY4741; *Mata; his3Δ1; leu2Δ0; met15Δ0; ura3Δ0; ARL3::GFP-HIS3MX6; naa30Δ::kanMX4*
TA255	BY4741; *Mata; his3Δ1; leu2Δ0; met15Δ0; ura3Δ0; TRM1::GFP-HIS3MX6*
TA264	BY4741; *Mata; his3Δ1; leu2Δ0; met15Δ0; ura3Δ0; TRM1::GFP-HIS3MX6; naa30Δ::kanMX4*
TA101	BY4741; *Mata; his3Δ1; leu2Δ0; met15Δ0; ura3Δ0; SEC18::GFP-HIS3MX6*
TA114	BY4741; *Mata; his3Δ1; leu2Δ0; met15Δ0; ura3Δ0; SEC18::GFP-HIS3MX6; naa30Δ::kanMX4*
TA258	BY4741; *Mata; his3Δ1; leu2Δ0; met15Δ0; ura3Δ0; SLY41::GFP-HIS3MX6*
TA285	BY4741; *Mata; his3Δ1; leu2Δ0; met15Δ0; ura3Δ0; SLY41::GFP-HIS3MX6; naa30Δ::kanMX4*
TA134	BY4741; *Mata; his3Δ1; leu2Δ0; met15Δ0; ura3Δ0; NUP157::GFP-HIS3MX6*
TA154	BY4741; *Mata; his3Δ1; leu2Δ0; met15Δ0; ura3Δ0; NUP157::GFP-HIS3MX6; naa30Δ::kanMX4*
TA126	BY4741; *Mata; his3Δ1; leu2Δ0; met15Δ0; ura3Δ0; RRN11::GFP-HIS3MX6*
TA139	BY4741; *Mata; his3Δ1; leu2Δ0; met15Δ0; ura3Δ0; RRN11::GFP-HIS3MX6; naa30Δ::kanMX4*
TA308	BY4741; *Mata; his3Δ1; leu2Δ0; met15Δ0; ura3Δ0; RFC2::GFP-HIS3MX6*
TA311	BY4741; *Mata; his3Δ1; leu2Δ0; met15Δ0; ura3Δ0; RFC2::GFP-HIS3MX6; naa30Δ::kanMX4*
TA127	BY4741; *Mata; his3Δ1; leu2Δ0; met15Δ0; ura3Δ0; YMR31::GFP-HIS3MX6*
TA159	BY4741; *Mata; his3Δ1; leu2Δ0; met15Δ0; ura3Δ0; YMR31::GFP-HIS3MX6; naa30Δ::kanMX4*
TA129	BY4741; *Mata; his3Δ1; leu2Δ0; met15Δ0; ura3Δ0; PDA1::GFP-HIS3MX6*
TA145	BY4741; *Mata; his3Δ1; leu2Δ0; met15Δ0; ura3Δ0; PDA1::GFP-HIS3MX6; naa30Δ::kanMX4*
TA131	BY4741; *Mata; his3Δ1; leu2Δ0; met15Δ0; ura3Δ0; GLR1::GFP-HIS3MX6*
TA147	BY4741; *Mata; his3Δ1; leu2Δ0; met15Δ0; ura3Δ0; GLR1::GFP-HIS3MX6; naa30Δ::kanMX4*
TA297	BY4741; *Mata; his3Δ1; leu2Δ0; met15Δ0; ura3Δ0; TMA20::GFP-HIS3MX6*
TA298	BY4741; *Mata; his3Δ1; leu2Δ0; met15Δ0; ura3Δ0; TMA20::GFP-HIS3MX6; naa30Δ::kanMX4*
TA128	BY4741; *Mata; his3Δ1; leu2Δ0; met15Δ0; ura3Δ0; LRG1::GFP-HIS3MX6*
TA142	BY4741; *Mata; his3Δ1; leu2Δ0; met15Δ0; ura3Δ0; LRG1::GFP-HIS3MX6; naa30Δ::kanMX4*
TA130	BY4741; *Mata; his3Δ1; leu2Δ0; met15Δ0; ura3Δ0; BEM1::GFP-HIS3MX6*
TA146	BY4741; *Mata; his3Δ1; leu2Δ0; met15Δ0; ura3Δ0; BEM1::GFP-HIS3MX6; naa30Δ::kanMX4*
TA262	BY4741; *Mata; his3Δ1; leu2Δ0; met15Δ0; ura3Δ0; PXL1::GFP-HIS3MX6*
TA274	BY4741; *Mata; his3Δ1; leu2Δ0; met15Δ0; ura3Δ0; PXL1::GFP-HIS3MX6; naa30Δ::kanMX4*
TA261	BY4741; *Mata; his3Δ1; leu2Δ0; met15Δ0; ura3Δ0; TGL1::GFP-HIS3MX6*
TA272	BY4741; *Mata; his3Δ1; leu2Δ0; met15Δ0; ura3Δ0; TGL1::GFP-HIS3MX6; naa30Δ::kanMX4*
TA132	BY4741; *Mata; his3Δ1; leu2Δ0; met15Δ0; ura3Δ0; PMT7::GFP-HIS3MX6*
TA150	BY4741; *Mata; his3Δ1; leu2Δ0; met15Δ0; ura3Δ0; PMT7::GFP-HIS3MX6; naa30Δ::kanMX4*
TA300	BY4741; *Mata; his3Δ1; leu2Δ0; met15Δ0; ura3Δ0; YMR31::GFP-HIS3MX6; pYX142-mtRFPm*
TA302	BY4741; *Mata; his3Δ1; leu2Δ0; met15Δ0; ura3Δ0; YMR31::GFP-HIS3MX6; naa30Δ::kanMX4; pYX142-mtRFPm*
TA304	BY4741 *Mata; his3Δ1; leu2Δ0; met15Δ0; ura3Δ0; PDA1::GFP-HIS3MX6; pYX142-mtRFPm*
TA306	BY4741; *Mata; his3Δ1; leu2Δ0; met15Δ0; ura3Δ0; PDA1::GFP-HIS3MX6; naa30Δ::kanMX4; pYX142-mtRFPm*
